# Synaptic and nonsynaptic plasticity approximating probabilistic inference

**DOI:** 10.3389/fnsyn.2014.00008

**Published:** 2014-04-08

**Authors:** Philip J. Tully, Matthias H. Hennig, Anders Lansner

**Affiliations:** ^1^Department of Computational Biology, Royal Institute of Technology (KTH)Stockholm, Sweden; ^2^Stockholm Brain Institute, Karolinska InstituteStockholm, Sweden; ^3^School of Informatics, Institute for Adaptive and Neural Computation, University of EdinburghEdinburgh, UK; ^4^Department of Numerical Analysis and Computer Science, Stockholm UniversityStockholm, Sweden

**Keywords:** Bayes' rule, synaptic plasticity and memory modeling, intrinsic excitability, naïve Bayes classifier, spiking neural networks, Hebbian learning

## Abstract

Learning and memory operations in neural circuits are believed to involve molecular cascades of synaptic and nonsynaptic changes that lead to a diverse repertoire of dynamical phenomena at higher levels of processing. Hebbian and homeostatic plasticity, neuromodulation, and intrinsic excitability all conspire to form and maintain memories. But it is still unclear how these seemingly redundant mechanisms could jointly orchestrate learning in a more unified system. To this end, a Hebbian learning rule for spiking neurons inspired by Bayesian statistics is proposed. In this model, synaptic weights and intrinsic currents are adapted on-line upon arrival of single spikes, which initiate a cascade of temporally interacting memory traces that locally estimate probabilities associated with relative neuronal activation levels. Trace dynamics enable synaptic learning to readily demonstrate a spike-timing dependence, stably return to a set-point over long time scales, and remain competitive despite this stability. Beyond unsupervised learning, linking the traces with an external plasticity-modulating signal enables spike-based reinforcement learning. At the postsynaptic neuron, the traces are represented by an activity-dependent ion channel that is shown to regulate the input received by a postsynaptic cell and generate intrinsic graded persistent firing levels. We show how spike-based Hebbian-Bayesian learning can be performed in a simulated inference task using integrate-and-fire (IAF) neurons that are Poisson-firing and background-driven, similar to the preferred regime of cortical neurons. Our results support the view that neurons can represent information in the form of probability distributions, and that probabilistic inference could be a functional by-product of coupled synaptic and nonsynaptic mechanisms operating over several timescales. The model provides a biophysical realization of Bayesian computation by reconciling several observed neural phenomena whose functional effects are only partially understood in concert.

## Introduction

Bayesian inference provides an intuitive framework for how the nervous system could internalize uncertainty about the external environment by optimally combining prior knowledge with information accumulated during exposure to sensory evidence. Although probabilistic computation has received broad experimental support across psychophysical models describing the perceptual and motor behavior of humans (Wolpert and Körding, 2004; Knill, 2005; Tassinari et al., 2006), it is nevertheless an open theoretical issue at which level of detail within the neural substrate it should be embedded (Knill and Pouget, 2004). Furthermore, synthesizing a probabilistic perspective with experimental data is a decidedly non-trivial task (Doya et al., 2007). Realizations of disparate phenomena occurring within the cortical circuitry have been hypothesized to represent viable coding schemes for such Bayesian principles, including single neurons (Denève, 2008a,b), neural population responses (Ma et al., 2006; Boerlin and Denève, 2011), specifically within the parietal (Yang and Shadlen, 2007) and prefrontal (D'Acremont et al., 2013) cortices, activation levels in the visual cortical hierarchy (Carpenter and Williams, 1995; Rao and Ballard, 1999; Summerfield and Koechlin, 2008; Berkes et al., 2011), long-term synaptic plasticity (Soltani and Wang, 2009), and short-term synaptic plasticity (Pfister et al., 2010; Stevenson et al., 2010). However, inductive frameworks notoriously tend to impose restrictions about when learning should occur (if at all) and account for a fraction of the diversity in physiological processes whose given anatomical granularity is otherwise arbitrary.

We propose a spike-based extension of the Bayesian Confidence Propagation Neural Network (BCPNN) plasticity rule (Lansner and Ekeberg, 1989; Lansner and Holst, 1996) to address these issues. In this model, storage and retrieval are enabled by gathering statistics about neural input and output activity. Synaptic weights are effectively inferred using Bayes' rule by incrementally (Sandberg et al., 2002) estimating confidence of feature observations from the input and posterior probabilities of outcome from the output. Weight modification depends on the temporal integration of spikes on different time scales using local synaptic traces, whose time courses are inspired by the cascade of events involved in the induction and maintenance of Hebbian plasticity. These traces estimate probabilities that determine synaptic weights and biases, which enable postsynaptic IAF neurons to signal through their relative spike rates the posterior likelihood of activation upon presentation of evidence in the form of presynaptic spiking.

The model suggests a non-redundant role for the presence of and interaction between a range of different processes in approximating probabilistic computation. Spike-based BCPNN can learn the temporal dimension of the input through modulation of its synaptic trace kinetics. Different spike timing-dependent plasticity (STDP) (Markram et al., 1997; Bi and Poo, 1998; Froemke and Dan, 2002) kernels can be predicted that promote learning forwards or backwards through time. Crucially, a unimodal stationary distribution of synaptic weights naturally follows from the learning rule due to an inherent multiplicative decay of the weights over long time scales, generating convergence behavior that is functionally reminiscent of synaptic scaling (Turrigiano et al., 1998). A global neuromodulatory signal is shown to provide information about rewards or expected rewards (Florian, 2007). The bias term, which represents prior confidence pending input evidence, is recast here as a Ca^2+^ sensitive, activity-dependent K^+^ current whose functional outcome resembles long-term potentiation of intrinsic excitability (LTP-IE) (Cudmore and Turrigiano, 2004). This interpretation allows us to replicate experiments from cortical neurons that suggested these factors could underlie graded persistent changes in firing levels (Egorov et al., 2002).

Increased efforts have focused on identifying the interplay of multiple synaptic (Keck et al., 2012) and even nonsynaptic (Habenschuss et al., 2012; Nessler et al., 2013; Savin et al., 2014) empirically grounded phenomena that could be relevant for learning and inference. In spike-based BCPNN, the use of evolving traces that coalesce to estimate probabilistic quantities complements these approaches by offering a conceivable way in which molecular events, which are known to span across different plasticity modalities (Daoudal and Debanne, 2003) and time scales (Tetzlaff et al., 2012), could be interconnected through latent probabilistic operations. The proposed model yields insights into how local and global computations, viewed through the lens of Bayes' rule, could accommodate a complex mixture of dynamics thought to be relevant for information processing in neocortex.

## Materials and methods

### Derivation of a probabilistic learning rule

Theoretical underpinnings described in this section are not intended to be a novel contribution, but are briefly included for completeness (Lansner and Ekeberg, 1989; Lansner and Holst, 1996). Consider a paradigm in which learning and recall are probabilistically grounded, associative memory mechanisms. According to BCPNN, computational units representing stochastic events have an associated activation state reflected by a real value between 0 and 1. This corresponds to the probability of that event, given observed events, which are represented by other active units. In spike-based BCPNN, units are viewed as local populations of 30 spiking neurons (Peters and Yilmaz, 1993), i.e., minicolumns, that have similar receptive fields and are highly connected and coactive (Mountcastle, 1997; Yoshimura et al., 2005; Bathellier et al., 2012). Corresponding levels of activation for these minicolumns are represented by their average spike rate.

Starting from Bayes' rule for relating the conditional probabilities of two random variables, observed firing rates collected from *n* presynaptic minicolumns *x*_1…*n*_, i.e., the evidence *P*(*x*_1…*n*_), can better inform the firing probabilities of neurons in the postsynaptic minicolumn *y*_*j*_, i.e., the prior *P*(*y_j_*):

(1) $$P(y_{j}|x_{1\ldots n})=P(y_{j})\frac{P(x_{1\ldots n}|y_{j})}{P(x_{1\ldots n})}$$

The described learning approach is tantamount to a naïve Bayes classifier that attempts to estimate the posterior probability distribution *P*(*y_j_*|*x*_1…*n*_) over a class (e.g., *y*_*j*_ = “animal”) realized by its observed attributes (e.g., *x*_*h*_ = “shape,” “color,” or “size”). By assuming conditional and unconditional independence between *x*_1…*n*_, Bayes' rule can be extended by:

(2) $$P(y_{j}|x_{1\ldots n})=P(y_{j})\frac{P(x_{1}|y_{j})}{P(x_{1})}\frac{P(x_{2}|y_{j})}{P(x_{2})}\ldots \frac{P(x_{n}|y_{j})}{P(x_{n})}$$

**Figure 1:**
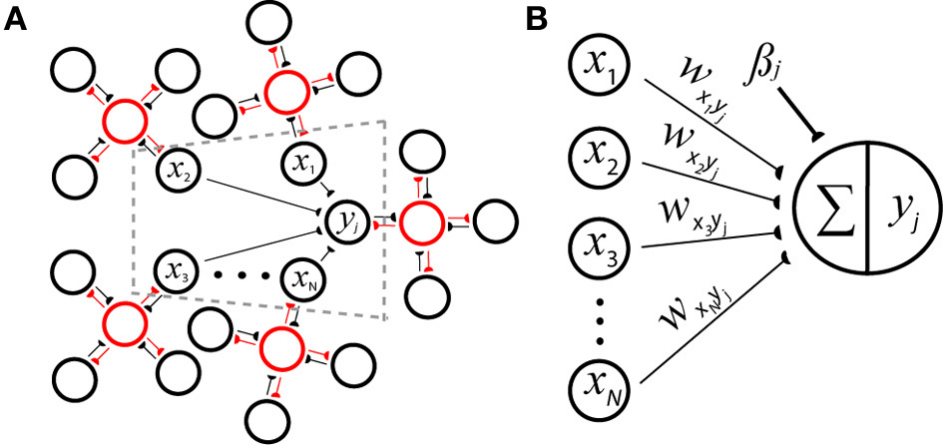
**Reconciling neuronal and probabilistic spaces using the spike-based BCPNN architecture for a postsynaptic minicolumn with activity *y_j_*. (A)** A cartoon of the derived network incorporates *H* = 5 hypercolumns each containing *n_h_* = 4 minicolumns that laterally inhibit each other (red lines) to perform a WTA operation via local inhibitory interneurons (red circles). The dotted gray area is represented by **B** in detail. **(B)** Weighted input rates *x*_1…*N*_ are summed and passed through a transfer function to determine the amount of output activation. Connections *w_x_i_y_j__* can be viewed as synaptic strengths (black lines, semicircles) or inverted directed acyclic graph edges representing the underlying generative model of a naïve Bayes classifier.

The assumption of independent marginals above is insignificant considering that the denominator of Equation 2 is identical for each *y_j_*. Thus, relative probabilistic ordering of classes remains intact, and probabilities can be recovered by normalizing *P*(*y_j_*|*x*_1…*n*_) to sum to 1. If we define each attribute *x_h_* as a discrete coded or as an interval coded continuous variable (e.g., *x*_*hi*_ = “blue,” “yellow,” or “pink” for *x_h_* = “color”), a modular network topology follows:

(3) $$P(y_{j}|x_{1\ldots n})=P(y_{j})\prod_{h\;=\;1}^{H}\sum_{i\;=\;1}^{n_{h}}\frac{P(x_{hi}|y_{j})}{P(x_{hi})}\pi_{x_{hi}}$$

in which *n*_*h*_ minicolumns are distributed into each of *H* hypercolumns (Figure 1A). Here, π*_x_hi__* represents relative activity or uncertainty of the attribute value *x*_*hi*_, and π*_x_hi__* = 1 indicates that attribute value *x*_*hi*_ was observed with maximal certainty. Equation 3 may instead be equivalently expressed as a sum of logarithms by:

(4) $$\log P(y_{j}|x_{1\ldots n})=\log P(y_{j})+\sum_{h\;=\;1}^{H}\log\left[\sum_{i\;=\;1}^{n_{h}}\frac{P(x_{hi}|y_{j})}{P(x_{hi})}\pi_{x_{hi}}\right]$$

Equation 4 states that contributions via connections from minicolumns in the same hypercolumn need to be summed before taking the logarithm, then summed again. Such an operation might be performed dendritically. More generally, the sum inside the logarithm can be approximated by one term through the elimination of index *h*, since there are significantly more hypercolumns than incoming synapses per neuron in mammalian neocortical networks. Considering the asymptotically large size and sparse connectivity of these networks, it is statistically unlikely that a specific hypercolumn would receive more than one incoming connection from any other hypercolumn.

Each hypercolumn is regarded as having normalized activity $\sum_{i\;=\;1}^{n_{h}}\pi_{x_{hi}}=1$, and such canonical connectivity schemes along with the winner-take-all (WTA) operations they imply are prevalent throughout neocortex (Douglas and Martin, 2004). Hence in analogy to neural transduction, a support value $s_{j}=\beta_{j}+\sum_{i\;=\;1}^{N}\pi_{x_{i}}w_{x_{i}y_{j}}$ can be calculated by iterating over the set of possible conditioning attribute values *N* = *Hn_h_* for *y_j_* with weight *w_x_i_y_j__* and bias β_*j*_ update equations (Figure 1B):

(5) $$\beta_{j}=\log P(y_{j})\;w_{x_{i}y_{j}}=\log\frac{P(x_{i}|y_{j})}{P(x_{i})}=\log\frac{P(x_{i},\;y_{j})}{P(x_{i})P(y_{j})}$$

Activity statistics are gathered during learning and their relative importance is evaluated and expressed as weights and biases. After Bayesian updating, probabilities are recovered by normalizing *P*(*y_j_*|*x*_1…*n*_) to sum to 1 over each *y_j_* by using an exponential transfer function since *s_j_* = log *P*(*y_j_*|*x*_1…*n*_):

(6) $$P(y_{j}|x_{1\ldots n})=\frac{e^{s_{j}}}{\sum_{i\;=\;1}^{n_{h}}e^{s_{i}}}$$

It is important to note that from this point onward, we refer to *w* and β as models of the incoming synaptic strength and excitability of a neuron. In the case where multiple synaptic boutons from a pre- to postsynaptic target neuron exist, they are represented here as a single synapse.

### Probabilistic inference performed with local synaptic traces

Spike-based BCPNN is based on memory traces implemented as exponentially weighted moving averages (EWMAs) (Roberts, 1959) of spikes, which were used to estimate *P_i_*, *P_j_*, and *P*_*ij*_ as defined above (Equation 5). Temporal smoothing corresponds to integration of neural activity by molecular processes and enables manipulation of these traces; it is a technique commonly implemented in synapse (Kempter et al., 1999) and neuron (Gerstner, 1995) models. EWMAs can ensure newly presented evidence is prioritized over previously learned patterns because as old memories decay, they are gradually replaced by more recent ones.

**Figure 2:**
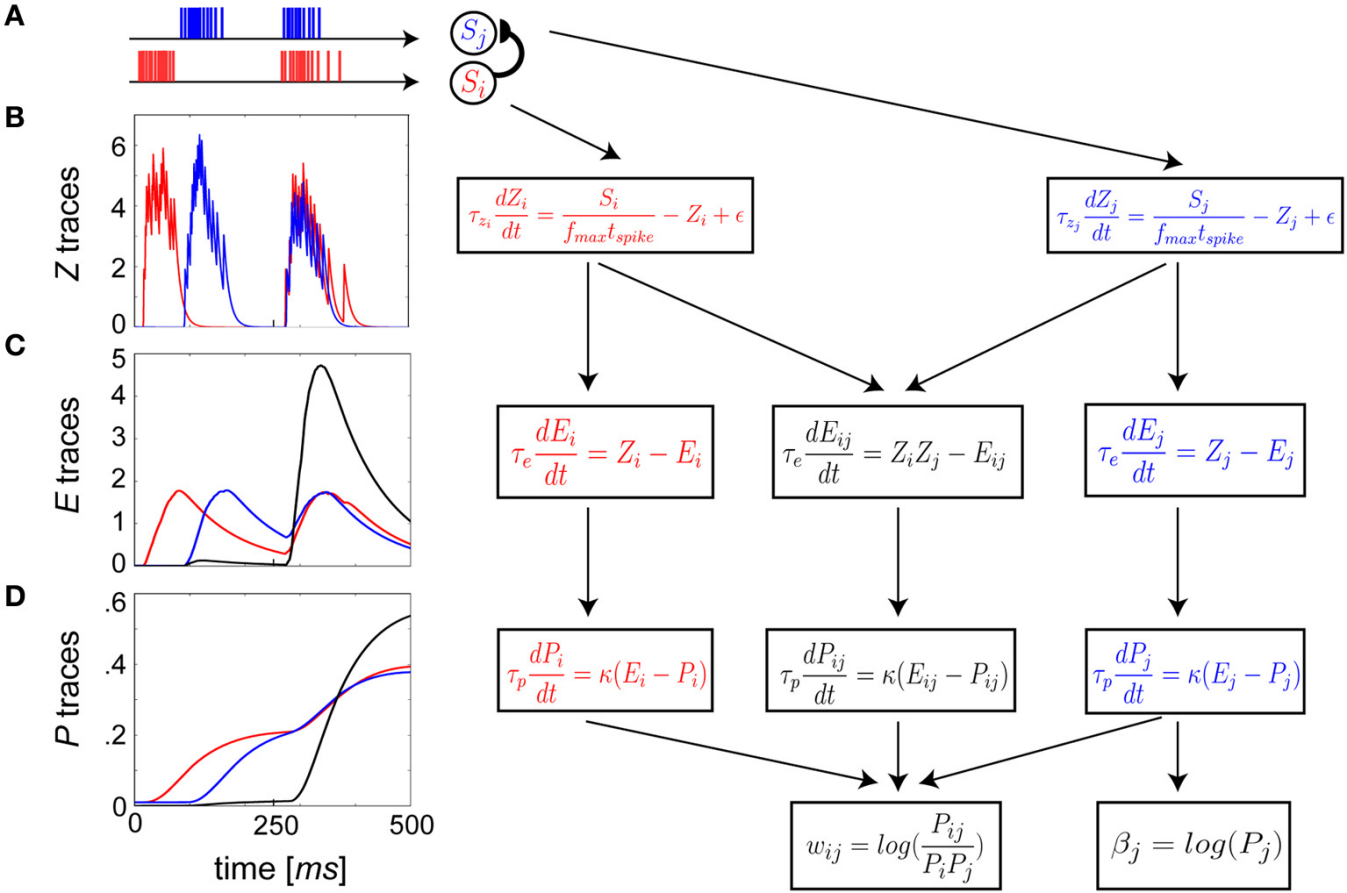
**Schematic flow of BCPNN update equations reformulated as spike-based plasticity. (A)** The *S_i_* pre- **(A–D**, red**)** and *S_j_* postsynaptic **(A–D**, blue**)** neuron spike trains are presented as arbitrary example input patterns. Each subsequent row **(B–D)** corresponds to a single stage in the EWMA estimate of the terms used in the incremental Bayesian weight update. **(B)** *Z* traces low pass filter input spike trains with τ*_z_i__* = τ*_z_j__*. **(C)** *E* traces compute a low pass filtered representation of the *Z* traces at time scale τ_*e*_. Co-activity now enters in a mutual trace **(C,D**, black**)**. **(D)** *E* traces feed into *P* traces that have the slowest plasticity and longest memory, which is established by τ_*p*_.

The dynamics governing the differential equations of the learning rule with two input spike trains, *S_i_* from presynaptic neuron *i* and *S_j_* from postsynaptic neuron *j*, are illustrated in Figure 2A. A three-stage EWMA procedure (Figures 2B–D) was adopted, the time constants of which were chosen to have a phenomenological mapping to key plasticity-relevant changes within signal transduction pathways that occur during learning.

The *Z_i_* and *Z_j_* traces had the fastest dynamics (Figure 2B), and were defined as

(7) $$\tau_{z_{i}}\frac{dZ_{i}}{dt}\text{​}=\text{​}\frac{S_{i}}{f_{\max}\;t_{spike}}\text{​}-\text{​}Z_{i}+\varepsilon \;\tau_{z_{j}}\frac{dZ_{j}}{dt}\text{​}=\text{​}\frac{S_{j}}{f_{\max}\;t_{spike}}\text{​}-\text{​}Z_{j}+\varepsilon$$

which filtered pre- and postsynaptic activity with time constants τ*_z_i__*, τ*_z_j__* ≈ 5–100 ms to match rapid Ca^2+^ influx via NMDA receptors or voltage-gated Ca^2+^ channels (Lisman, 1989; Bliss and Collingridge, 1993). These events initiate synaptic plasticity and can determine the time scale of the coincidence detection window for LTP induction (Markram et al., 1997).

We assumed that each neuron could maximally fire at *f*_max_ Hz and minimally at ϵ Hz, which represented absolute certainty and doubt about the evidential context of the input. Relative uncertainty was represented by firing levels between these bounds. Since every spike event had duration *t_spike_* ms, normalizing each spike by *f*_max_ *t*_*spike*_ meant that it contributed an appropriate proportion of overall probability in a given unit of time by making the underlying *Z* trace ≈1. This established a linear transformation between probability space ∈ {ϵ, 1} and neuronal spike rate ∈ {ϵ, *f*_max_}. Placing upper and lower bounds on firing rates was reasonable given physiologically relevant firing rates of cortical pyramidal neurons (Abeles, 1991).

The *Z* traces were passed on to the *E* or eligibility traces (Klopf, 1972), which evolved according to (Figure 2C):

(8) $$\tau_{e}\frac{dE_{i}}{dt}=Z_{i}-E_{i}\;\tau_{e}\frac{dE_{j}}{dt}=Z_{j}-E_{j}\;\tau_{e}\frac{dE_{ij}}{dt}=Z_{i}Z_{j}-E_{ij}$$

At this stage of the EWMAs, a separate equation was introduced to track coincident activity from the *Z* traces. Eligibility traces have been used extensively to simulate delayed reward paradigms in previous models (Florian, 2007; Izhikevich, 2007), and are viewed as a potential neural mechanism underlying reinforcement learning (Pawlak et al., 2010). They enabled simultaneous pre-post spiking to trigger a buildup of activity in the *E* traces, which could then be eligible for externally driven neuromodulatory intervention. The time constant τ_*e*_ ≈ 100–1000 ms was assumed to represent one of the downstream cellular processes that could interact with increased intracellular Ca^2+^ concentrations, such as CaMKII activation (Fukunaga et al., 1993). Creation of a decaying tag for each pre-post activated synapse for delivery of a specific marker that can be targeted for future plasticity-associated protein trafficking (Frey and Morris, 1997) has also been hypothesized to provide an intermediary step in the transition from early to late phase LTP.

*E* traces were subsequently passed on to the *P* traces (Figure 2D). Gene expression, protein synthesis and protein capture are cellular processes that mediate LTP maintenance and long-term memory formation (Nguyen et al., 1994; Frey and Morris, 1997). They tend to be activated in late phase LTP by elevated levels of Ca^2+^ dependent protein kinases, akin to activation in the *P* trace dynamics originating from sustained activation in the *E* traces:

(9) $$\tau_{p}\frac{dP_{i}}{dt}=\kappa (E_{i}-P_{i})\;\tau_{p}\frac{dP_{j}}{dt}=\kappa (E_{j}-P_{j})\;\tau_{p}\frac{dP_{ij}}{dt}=\kappa (E_{ij}-P_{ij})$$

Since these processes tend to exhibit highly variable timescales lasting anywhere from several seconds up to potentially days or months (Abraham, 2003), we simply imposed τ*_z_i__*, τ*_z_j__* < τ_*e*_ < τ_*p*_, but typically used τ_*p*_ ≈ 10 s for the sake of conciseness in simulations. Directly regulating the learning rate, parameter κ ∈ [0, ∞] represented the action of an endogenous neuromodulator, e.g., dopamine (Schultz et al., 1997), that signaled the relevance of recent synaptic events. The *P* trace is considered a versatile process tied closely to the nature of the task at hand by a globally applied κ (Schultz et al., 1997). Recently stored correlations were propagated when κ ≠ 0 and no weight changes take place when κ = 0. Although we show through simulations how delayed reward could be implemented with *E* traces, they are not required for inference, and having τ_*e*_ approach 0 would not undermine any of the results presented here.

Probabilities were ultimately fed into the final learning rule update equations (Equation 5) used to compute β_*j*_ and *w*_*ij*_:

(10) $$\beta_{j}=\log(P_{j})\;w_{ij}=\log\frac{P_{ij}}{P_{i}P_{j}}$$

**Figure 3:**
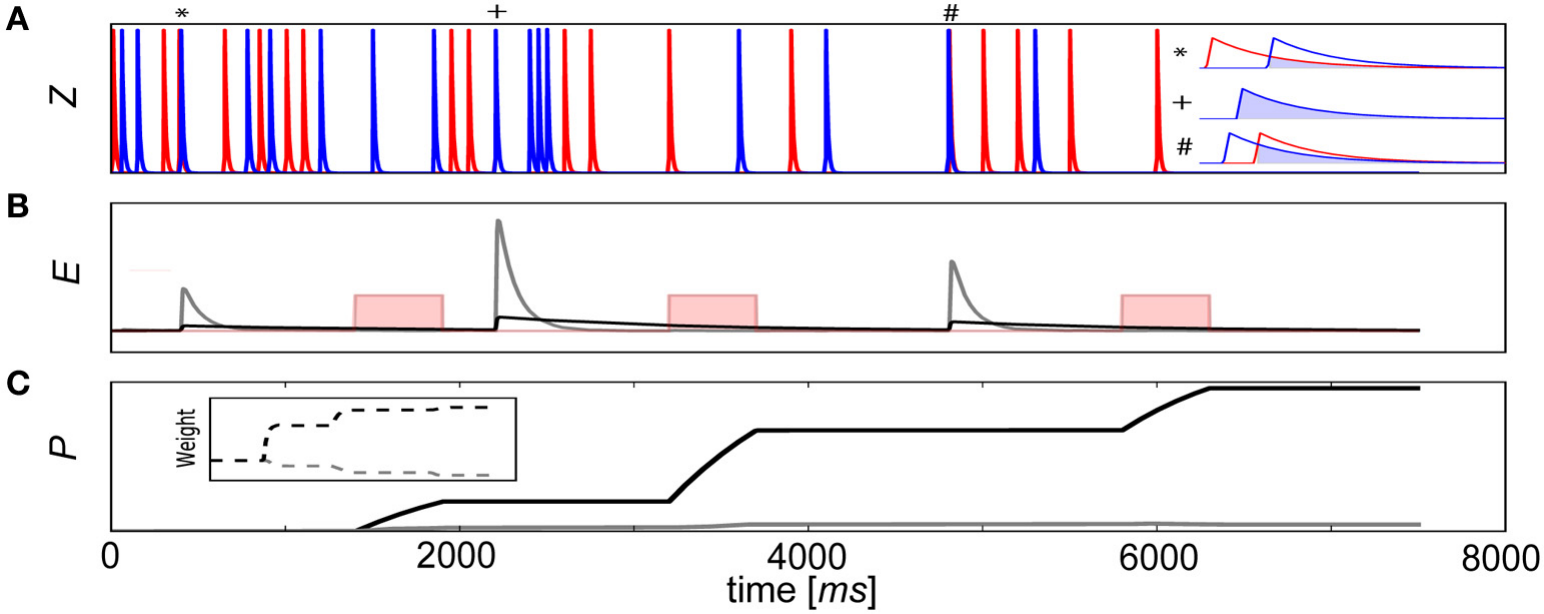
**Delayed reward learning using *E* traces. (A)** A pair of neurons fire randomly and elicit changes in the pre- (red) and postsynaptic (blue) *Z* traces of a BCPNN synapse connecting them. Sometimes by chance (pre before post^*^, synchronous^+^, post before pre^#^), the neurons fire coincidentally and the degree of overlap of their *Z* traces (inset, light blue), regardless of their order of firing, is propagated to the mutual eligibility trace *E*_*ij*_. **(B)** A reward (pink rectangular function, not to scale) is delivered as external supervision. Resulting *E* traces are indicated (gray line, τ_*e*_ = 100 ms and black line, τ_*e*_ = 1000 ms). **(C)** Behavior of color corresponding *P*_*ij*_ traces and weights (inset) depends on whether or not the reward reached the synapses in ample time.

To illustrate this process, a learning scheme involving delayed rewards is depicted with a pair of connected neurons (Figure 3A). In this example, a reward was delivered 1–2 s after coincident activity (Waelti et al., 2001) for 500 ms (Gonon, 1997) to reinforce deserving stimuli. If τ_*e*_ was too small or positive reward κ arrived after the *E* trace had decayed to baseline (Figure 3B), no signal was propagated to the *P* traces. As a result, the corresponding *P*_*ij*_ trace and weight remained unchanged. However, if the *E* trace was sufficiently large such that there was an overlap with κ, the strength of the synapse grew and associative learning transpired (Figure 3C). Although only one connection *w*_*ij*_ is depicted in this example, κ would be modulated in the same way for all synapses in the network context, typical of dopaminergic neuron release characteristics (Waelti et al., 2001).

### Leaky integrate-and-fire neuron model

Model spikes are generated using NEST version 2 (Gewaltig and Deismann, 2007). An IAF neuron with alpha function-shaped postsynaptic conductance, NEST model “iaf_cond_alpha” (Kuhn et al., 2004), is amended to account for the bias term β (Equation 10). It enters the sub-threshold voltage *V_m_* equation of the postsynaptic neuron according to:

(11) $$\begin{array}{l} -C_{m}\frac{dV_{m}}{dt}=g_{L}(V_{m}-E_{L})+\sum_{i=1}^{n}g_{ex,\;i}(V_{m}-E_{ex,\;i})\\ \;+\sum_{i=1}^{n}g_{inh,\;i}(V_{m}-E_{inh,\;i})+\phi I_{\beta } \end{array}$$

When threshold *V*_*th*_ is reached (*V_m_* ≥ *V_th_*) a spike is generated and *V_m_* is held to the reset potential *V*_*res*_ for *t*_*ref*_ ms representing the absolute refractory period. The total current flow across the membrane is determined by the membrane capacitance *C_m_*, the leak reversal potential *E*_*L*_, excitatory *E*_*ex*_ and inhibitory *E*_*inh*_ reversal potentials, the leak conductance *g*_*L*_, excitatory *g*_*ex*_ and inhibitory *g*_*inh*_ synaptic conductances, and *I*_β_ that is scaled to represent an activity-dependent current quantity by ϕ. Postsynaptic conductances *g*_*ex*_ and *g*_*inh*_ are modified by the occurrence of an excitatory or inhibitory input event from one of the *n* presynaptic neurons at time *t_s_* by:

(12) $$g_{ex|inh,i}(t)=g_{\max}w_{ij}\frac{t-t_{s}-d}{\tau_{ex|inh}}e^{\frac{1-(t-t_{s}-d)}{\tau_{ex|inh}}}$$

This enables *g*_*ex*_ or *g*_*inh*_ to rise with finite duration τ_*ex*_ or τ_*inh*_ to its peak conductance *g*_max_ at time *t* − *t_s_* − *d* = τ_*ex*_ or τ_*inh*_, where *d* is the transmission delay.

IAF neurons offer an analytically convenient form for describing rate of firing dependent upon quantifiable measures of *V_m_*. We will show in the Results that the input-output relationship in a background driven regime is particularly suited for Bayesian computations (Equation 6). If we consider an IAF neuron as it receives excitatory synaptic drive λ_*ex*_ = *n_ex_f_ex_w_ex_τ_ex_e* from *n*_*ex*_ Poisson processes spiking at *f*_*ex*_ Hz with weights $w_{ex}=\sum_{i=1}^{n_{ex}}w_{ij}$, its mean firing rate *r* can be formulated according to Kuhn et al. (2004):

(13) $$r(\mu_{m},\;\sigma_{m})=\frac{1}{2\tau_{m}}\left[1-erf\left(\frac{V_{th}-\mu_{m}}{\sigma_{m}\sqrt{2}}\right)\right]$$

where τ_*m*_ = *C_m_*/(*g_L_* + λ_*ex*_) is the effective membrane time constant, *erf* is the error function, and the steady state mean μ_*m*_ and standard deviation σ_*m*_ of its *V_m_* are estimated by (Figure S1):

(14) $$\mu_{m}=\frac{E_{L}G_{L}+E_{ex}\lambda_{ex}}{G_{L}+\lambda_{ex}}\;\sigma_{m}=\sqrt{n_{ex}f_{ex}(2\tau_{m}+\tau_{ex}){\left[\frac{(E_{ex}-\mu_{m})\lambda_{ex}\tau_{m}}{2C_{m}(\tau_{m}+\tau_{ex})}\right]}^{2}}$$

In numerical simulations, neurons were stimulated by Poisson spike trains or correlated spike trains, the latter of which were generated using the Multiple Interaction Process (Kuhn et al., 2003) defined in NEST (“mip_generator”). For simulations where background activity was present, 30 input Poisson sources stimulated each neuron to control their background spike rate. The values of all synaptic and neuronal parameters used in numerical simulations are listed in Table 1.

**Table 1:** **When parameters are not explicity listed in the text, they are interleaved below, following (Nordlie et al., 2009)**.

**(A) MODEL SUMMARY**	
Neuron model	Leaky IAF
Synapse model	Conductance-based with *α*-shaped PSCs, plastic BCPNN synapses
Channel model	K^+^ channel
Input model	Fixed-rate Poisson spike trains
Measured quantities	Spike activity, connection strengths, biases, voltages
**(B) NEURON MODEL**	
Leaky IAF dynamics	Subthreshold membrane potential *V_m_* of neuron *j* with *n* inputs: $-C_{m}\frac{dV_{m}}{dt}=g_{L}(V_{m}-E_{L})+\sum_{i=1}^{n}g_{ex,i}(V_{m}-E_{ex,i})+\sum_{i=1}^{n}g_{inh,\;i}(V_{m}-E_{inh,\;i})+\phi I_{\beta }$
	Spiking: If *V*_*m*_ ≥ *V_th_* spike generated and *V*_*m*_ held at *V*_*res*_ for *t*_*ref*_ ms
Parameters	*C*_*m*_ = 250 pF membrane capacitance
	*g*_*L*_ = 16.67 nS leak conductance
	*E*_*L*_ = −70 mV leak reversal potential
	*E*_*ex*_ = 0 mV excitatory reversal potential
	*E*_*inh*_ = −75 mV inhibitory reversal potential
	ϕ = 50 pA current scaling factor Figures 8–10, 0 pA otherwise
	*V*_*th*_ = −55 mV membrane voltage threshold
	*V*_*res*_ = −60 mV membrane reset potential
	*t*_*ref*_ = 2 ms refractory period
	*dt* = 0.1 ms time resolution
**(C) CHANNEL MODEL**	
Activity-dependent hyperpolarizing	K^+^/CAN current of neuron *j*, *I*_β*j*_ pA: $\tau_{p}\frac{dP_{j}}{dt}=\kappa (E_{j}-P_{j})$
	*I*_β*j*_ = ϕ β_*j*_ = ϕlog (*P_j_*)
	See Equation 8 for calculation of *E*_*j*_
Parameters	τ_*zj*_ = 10 ms *Z* trace time constant
	τ_*e*_ = 100 ms *E* trace time constant
	τ_*p*_ = 10000 ms *P* trace time constant
	*S*_*j*_ = 1 if spike, 0 if no spike
	*f*_max_ = 20 Hz, highest firing rate
	ϵ = 1/(*f*_max_τ_*p*_) Hz, lowest firing rate
	*t*_*spike*_ = 0.1 ms spike duration
	**(D) SYNAPSE MODEL**	
α-shape PSC dynamics	Excitatory *g*_*ex*_ and inhibitory *g*_*inh*_ conductance changes for postsynaptic neuron *j* with spike at time *t*_*s*_ by one of the *n* presynaptic neurons: $g_{ex|inh,\;i}(t)=g_{\max}w_{ij}\frac{t-t_{s}-d}{\tau_{ex|inh}}e^{\frac{1-(t-t_{s}-d)}{\tau_{ex|inh}}}$
BCPNN synapse	Synaptic strength between *i* and *j*, *w*_*ij*_ nS: $\tau_{p}\frac{dP_{i}}{dt}=\kappa (E_{i}-P_{i}),\tau_{p}\frac{dP_{j}}{dt}=\kappa (E_{j}-P_{j}),\tau_{p}\frac{dP_{ij}}{dt}=\kappa (E_{ij}-P_{ij})$
	$w_{ij}=\log\left(\frac{P_{ij}}{P_{i}P_{j}}\right)$
	See Equation 8 for calculation of *E*_*j*_, *E_j_* and *E*_*ij*_.
Parameters	τ_*zi*_ = 10 ms *Z* trace time constant
	τ_*e*_ = 100 ms *E* trace time constant
	τ_*p*_ = 10000 ms *P* trace time constant
	*g*_max_ = 2.0 nS peak conductance
	τ_*ex*_ = 0.2 ms α rise time for excitatory input
	τ_*inh*_ = 2 ms α rise time for inhibitory input
	*d* = 0.1 ms transmission delay
	κ = 1.0, 0.0 to freeze plasticity (Figure 10)
**(E) INPUT**	
Poisson generator	*n*_*ex*_ = 30 processes, independent per neuron
	*w*_*ex*_ = 10.75 nS per process
	*r*_*ex*_ rate, 0 < *r_ex_* < *f_max_*
**(F) MEASUREMENTS**	
*r* spike rate (spikes/second)	
*w*_*ij*_ synaptic weight between neurons *i* and *j* (nS)	
*V*_*m*_ membrane voltage of neuron *j* (mV)	
*I*_β_bias current magnitude (pA)	

## Results

We found that dynamical phenomena emerging from this mapping resembled processes that are thought to underlie learning and memory in cortical microcircuits. We first identify the synaptic and nonsynaptic correlates of this extension by studying ensuing spike dynamics accompanying the individual assumptions of the derivation, and then the functionally distinct computations are considered together in a network setting where we demonstrate a simple Bayesian inference task performed by spiking neurons.

### Validating spike-based BCPNN with previous implementations

As a proof of concept, we first sought to validate whether using EWMAs with input Poisson trains in spike-based BCPNN could reliably estimate learning outcomes of an abstract BCPNN where units had simple, exponentially smoothed binary activation patterns (Equation 5) (Sandberg et al., 2002). To demonstrate consistency, five patterns between two units (binary activations of 1 or 0) and two neurons (Poisson spike trains firing at *f*_max_ or ϵ Hz) were instantiated in ten consecutive 200 ms trials. In this setup, we set τ_*p*_ = 1000 ms by design to be less than this 2000 ms presented pattern duration.

**Figure 4:**
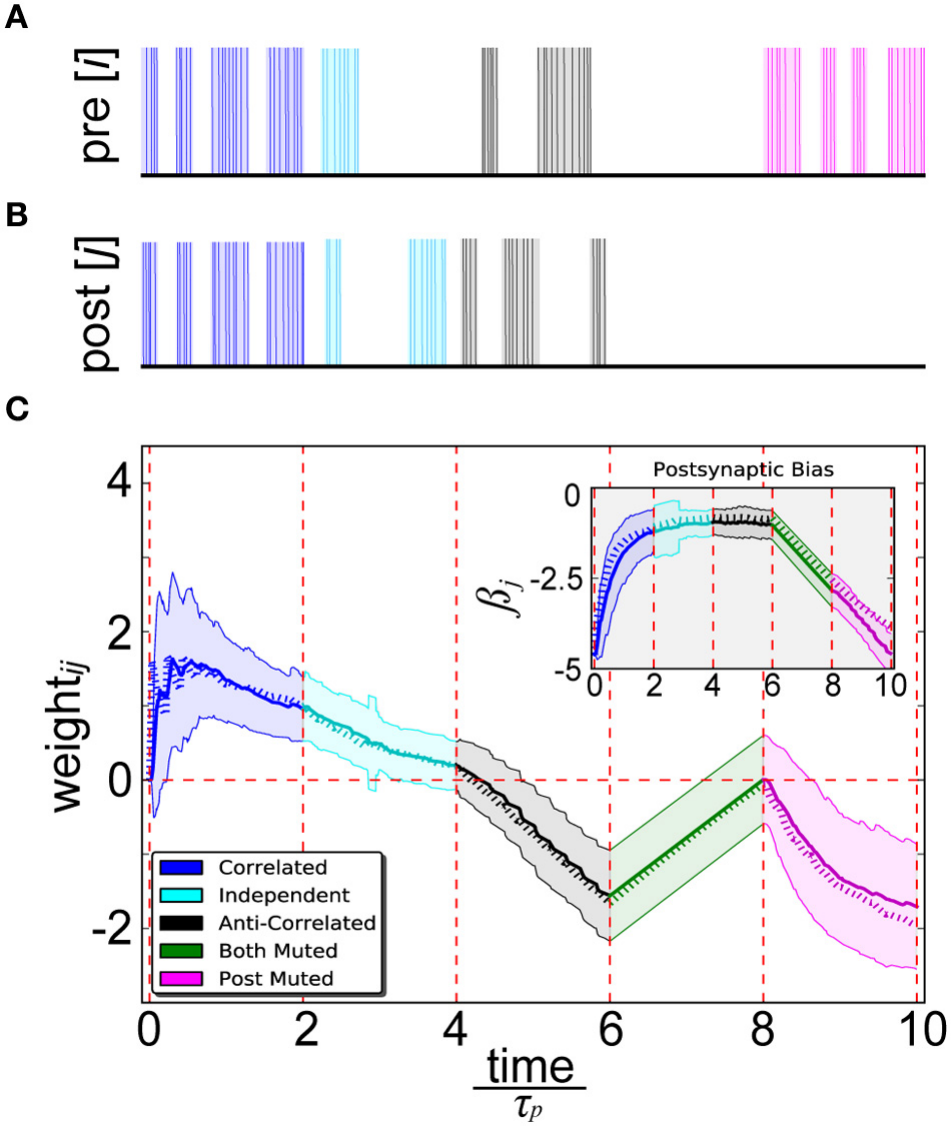
**Spike-based BCPNN estimates abstract BCPNN for different input patterns. (A)** Pre- and **(B)** postsynaptic input spike trains. Activation patterns (shaded rectangles) of abstract BCPNN units and corresponding Poisson spike trains (vertical bars) firing at *f*_max_ Hz elicited in IAF neurons are differentiated by color. **(C)** Weight and bias (inset) development under different protocol for the abstract (dotted) and spike-based (solid) versions of the learning rule. Spiking simulations were repeated 100 times and averaged, with standard deviations illustrated by the shaded regions.

By simultaneously presenting proportional unit activity and spiking patterns to the pre- (Figure 4A) and postsynaptic (Figure 4B) binary output units of abstract BCPNN and IAF neurons of spike-based BCPNN, a close correspondence between their resulting weight and bias trajectories was confirmed (Figure 4C). Five separate cases were tested in order to robustly sample statistical relationships among a diverse set of patterns. Correlated patterns meant both units/neurons were maximally or minimally active/firing in each trial, independent patterns denoted uniform sampling of active and inactive patterns for both neurons in each trial, anti-correlated patterns meant one was active and the other was inactive or vice-versa in each trial, both muted meant both were inactive in all trials, and post muted meant activity of the presynaptic neuron was uniformly sampled and the postsynaptic one was inactive in all trials.

We found some notable differences between spike-based BCPNN and other correlation-based learning rules. Instances in which neuron *i* was highly active and neuron *j* weakly active (and vice versa) led to a decay of *w*_*ij*_, which eventually turned negative. When *i* and *j* were both either highly or weakly active, *w*_*ij*_ increased because correspondingly active or correspondingly inactive patterns are indistinguishable from a probabilistic viewpoint. The increase of *w*_*ij*_ when *i* and *j* were both weakly active was linearly dependent upon the three exponentially decaying *P* traces (Equation 9), since they tended to decay toward ε in the absence of any input. When *i* and *j* were both highly active, learning was virtually instantaneous, or one-shot, since τ_*p*_ was short compared with the stimulus duration. Steady state trace dynamics were responsible for the eventual decay of positive weights over time, similar to the multiplicative enforcement of constraints previously proposed on theoretical grounds (Miller and Mackay, 1994). Importantly, this built-in compensatory mechanism was much slower than weight increases, otherwise its regulatory effects would have dampened any transient activity fluctuations that could have been relevant for information processing and memory.

### Plasticity dynamics of spike-based BCPNN

**Figure 5:**
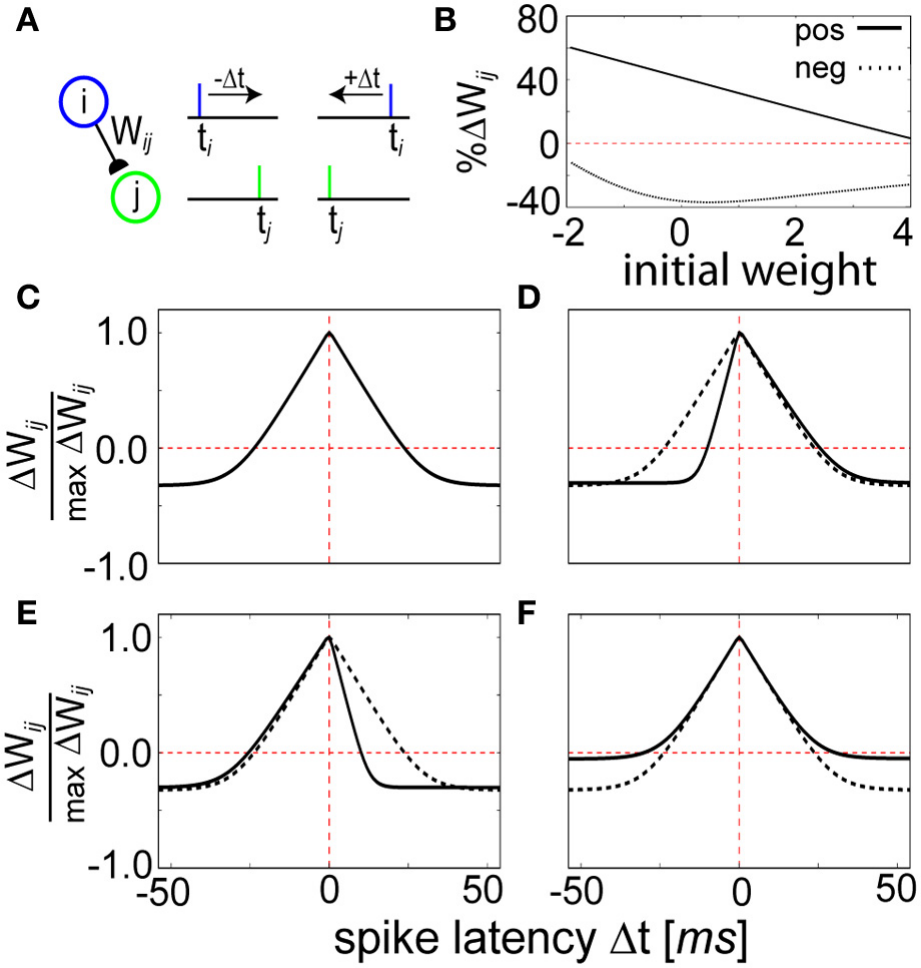
**STDP function curves are shaped by the *Z* trace time constants. (A)** Schematic representation of the STDP conditioning protocol. Each pre (blue)—post (green) pairing is repeated for each time difference Δ*t* = *t*_1_ − *t*_2_ illustrated in **(C–E)**. **(B)** Weight dependence for positive (Δ*t* = 0 ms, solid line) and negative (Δ*t* = 50 ms, dashed line) spike timings. Compare to Figure 5 of Bi and Poo (1998). **(C)** Relative change in peak synaptic amplitude using τ_*zi*_ = 5 ms, τ_*zj*_ = 5 ms, τ_*e*_ = 100 ms, and τ_*p*_ = 10000 ms. This curve is reproduced in **(D–F)** using dotted lines as a reference. **(D)** The width of the LTP window is determined by the magnitude of the *Z* trace time constants. When τ_*zj*_ is changed to 2 ms, the coincident learning window shifts right. **(E)** Instead when τ_*zi*_ is changed to 2 ms, it shifts left. Note that a decrease in τ_*zi*_ is thus qualitatively consistent with the canonical STDP kernel. **(F)** Changing the *P* trace time constant influences the amount of LTD. When τ_*p*_ is doubled to 20,000 ms, the learned correlations tend to decay at a slower rate.

The spiking setup allowed us to consider more detailed temporal aspects of plasticity beyond simple rate-modulated Poisson processes. First, we investigated how the temporal relationship between pre- and postsynaptic activity influenced expression of plasticity in our model. To evaluate the STDP properties of spike-based BCPNN, a canonical experimental protocol was simulated (Markram et al., 1997; Bi and Poo, 1998) by inducing pre- (*t_i_*) and postsynaptic (*t_j_*) spiking in IAF neurons shortly before or after one another 60 times at 1 Hz frequency without background activity (Figure 5A).

The strength of the weight changes were bidirectional and weight-dependent (Figure 5B), generally exhibiting LTP for tight values of Δ*t* = *t_i_* − *t_j_* and LTD for wider values of Δ*t* (Figure 5C). The shape of the learning window was dependent upon the parameters τ_*zi*_, τ_*zj*_, and τ_*p*_, defining the duration of the different memory traces in the model (see Materials and Methods). Manipulation of the *Z* trace time constants changed the width of the STDP window, and therefore τ_*zi*_ and τ_*zj*_ effectively regulated sensitivity to spike coincidence. Having τ_*zi*_ ≠ τ_*zj*_ generated an asymmetric weight structure that allowed for prioritization of pre-post timing (+Δ*t*) over post-pre timing (−Δ*t*, Figure 5D) and vice versa (Figure 5E). The LTD area shrank for a constant STDP window width when τ_*p*_ was increased because it induced a longer decay time for the *P* traces (Figure 5F), emphasizing a slowness in learning. Temporally symmetrical Hebbian learning was due to an increase of *P*_*ij*_ as a result of the amount of overlap between *P_i_* and *P_j_* (see Figure 2D). A similar form of LTP based on pre- and postsynaptic spike train overlap (Figure S2) has been shown for synapses in slices (Kobayashi and Poo, 2004).

### An emergent approach to the stability vs. competition dilemma

Long-term stability can be problematic for correlative learning rules (e.g., Figure 5C), since bounded Hebbian synapses destabilize plastic networks by maximally potentiating or depressing synapses. Additional mechanisms such as weight-dependent weight changes (van Rossum et al., 2000) or fine tuning of window parameters (Kempter and Gerstner, 2001; Babadi and Abbott, 2010) have been shown to be able to keep weights in check. In contrast, owing to its plasticity dynamics during on-line probability estimation, spike-based BCPNN naturally demonstrated weight dependence (Figure 5B) along with a stable unimodal equilibrium weight distribution when exposed to prolonged uncorrelated stimulation.

**Figure 6:**
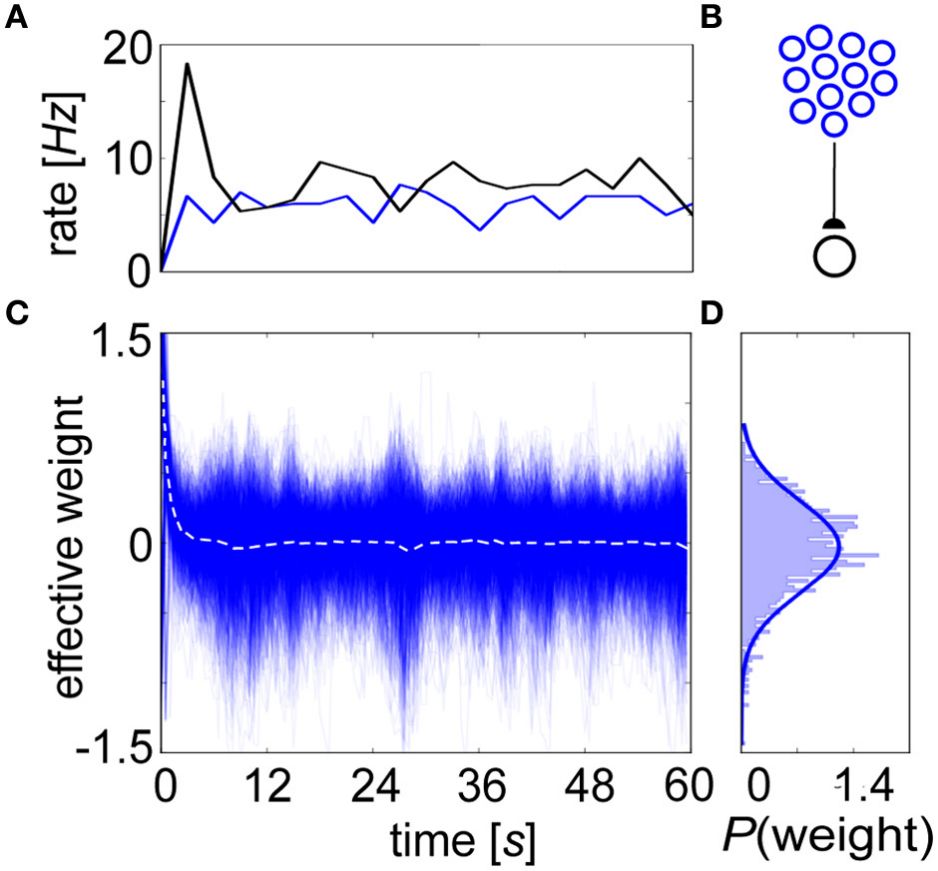
**The BCPNN learning rule exhibits a stable equilibrium weight distribution. (A)** Progression of averaged rates of firing (3 s bins) for the presynaptic (blue) and postsynaptic (black) neurons in the network. **(B)** Setup involves 1000 Poisson-firing presynaptic neurons that drive one postsynaptic cell. **(C)** The BCPNN synaptic strengths recorded every 100 ms (blue, dotted white line is their instantaneous mean) has an initial transient but then remains steady throughout the entire simulation despite deviation amongst individual weights within the equilibrium distribution. **(D)** BCPNN weight histogram plotted for the final time epoch is unimodal and approximately normally distributed (blue line, μ_0_ = 0.0 and σ_0_ = 0.38).

**Figure 7:**
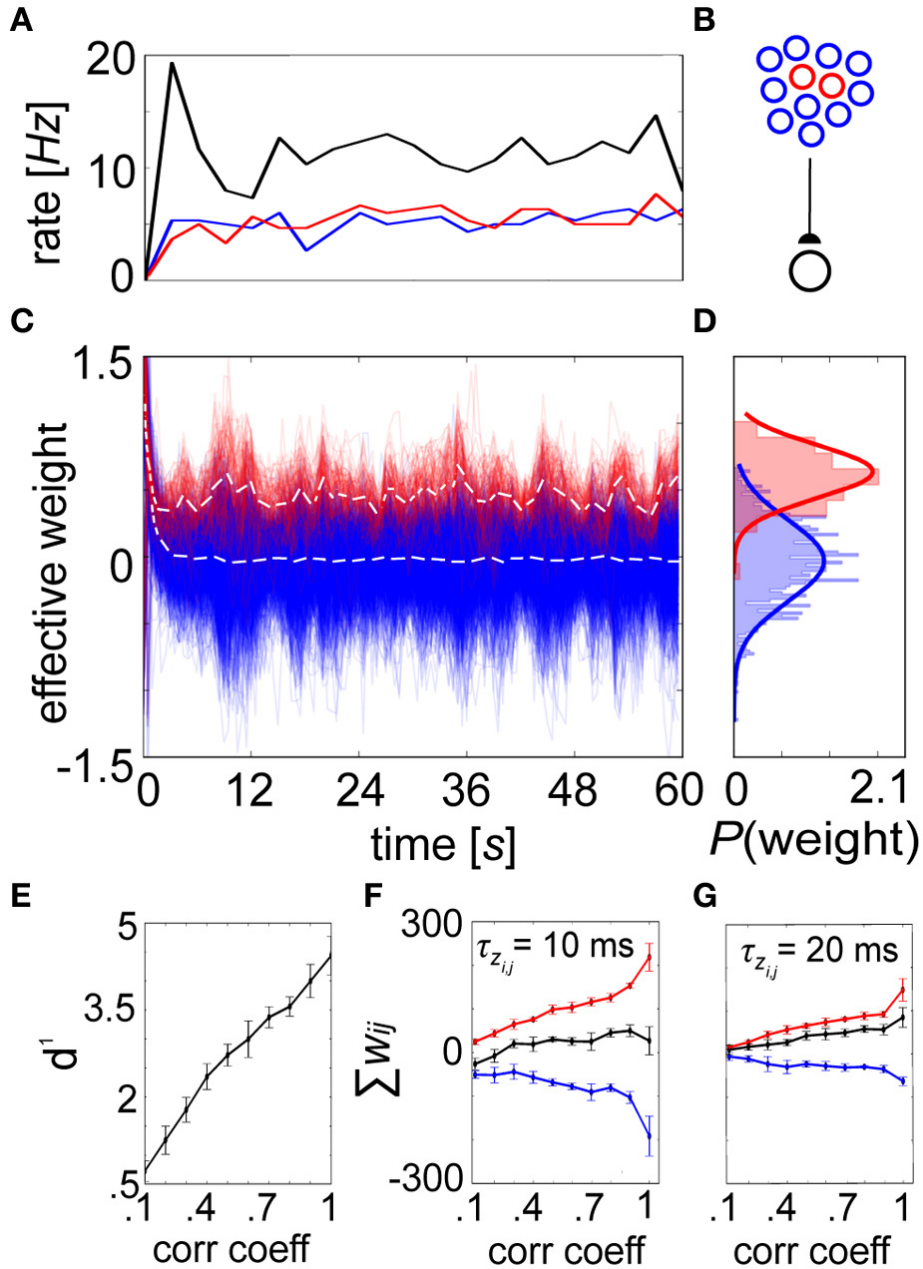
**A shift in the weight distribution of correlated neurons arises from structured input. (A)** Progression of averaged rates of firing (3 s bins) for the externally stimulated uncorrelated (blue) and correlated (pictured *C* = 0.2, red) presynaptic neurons, along with the postsynaptic (black) neuron they drive in the network. **(B)** Setup involves 900 uncorrelated and 100 correlated presynaptic neurons that drive one postsynaptic cell. **(C)** Synaptic strengths recorded every 100 ms from the correlated group gradually specialize over time vs. their uncorrelated counterparts, resulting in a change in the mean distribution of weights (white dotted lines for each, here *C* = 0.2). **(D)** Weight histograms plotted for the final time epoch are unimodal and approximately normally distributed (*C* = 0.2, μ_0_ = −0.03, μ_0_ = 0.34 and σ_0_ ≈ σ_+_ = 0.18). **(E)** The separation between these distributions is expressed as *d*^1^, which increases as a function of the input correlation coefficient. **(F)** Summed weights for the correlated (red), uncorrelated (blue), and combined (black) in the final epoch as a function of *C*. **(G)** Same as in **(F)** but with τ_*zi*_ and τ_*zj*_ increased by a factor of 2. In both instances, the combined weights remain relatively constant around *w*_*ij*_ = 0, although lower time constants induce more substantial differences between the correlated and uncorrelated weights. Error bars depict the standard deviation gathered from 50 repeated trials.

We conducted equilibrium experiments (Figures 6, 7) using spike-based BCPNN synapses in which each of their mean stationary weight distributions were shifted upwards by the lowest possible allowed weight. This subtrahend was calculated from Equation 10, log(ϵ^2^/0.5^2^) = log(4ε^2^), or the log minimum *P*_*ij*_ = ϵ^2^ (no co-activity) divided by maximum *P_i_*P_*j*_ = 0.5^2^ (both pre- and post-neurons are active half of the time) trace values. Although this normalization would not occur biologically, it was necessary for displaying true equilibrium weight values because the average weight distribution ≈ 0 after τ_*p*_ ms due to *P* trace decay, and zero-valued average weights would have mitigated any postsynaptic response in the absence of background input. To demonstrate stability, a postsynaptic neuron is shown steadily firing at an average of 7 Hz when innervated by 1000 presynaptic input neurons each producing 5 Hz Poisson spike trains due to background activity (Figure 6A). Given this setup (Figure 6B), the evolution of the renormalized synaptic weights during this period settled around 0 (Figure 6C).

This behavior can be understood by investigating the *P* traces. Initially, both *P_i_* and *P_j_* increased as presynaptic input elicited postsynaptic spiking, growing the value of the denominator from Equation 10. In the numerator, the mutual trace *P*_*ij*_ built up as well, and there was an eventual convergence in the *P* traces to $P_{i}P_{j}=\sqrt{P_{ij}}$ after an elapsed time τ_*p*_. Because both neurons fired together, the learning rule initially enhanced their connection strength, creating an initial transient output rate excursion. But as input persisted such that pre- and postsynaptic neurons continued firing at constant rates, correlations were eventually lost due to *P* trace decay. Statistically speaking, the signals emitted by the two neurons were indistinguishable over long timescales. The steady state of the weights ended up approximately Gaussian distributed around the quotient log(1) ≈ 0 (Figure 6D), independent of the approximate rates for the pre- and postsynaptic neurons. This stability was robust to the choice of time constants, given relatively constant pre- and postsynaptic firing rates.

But presence of a unimodal equilibrium weight distribution alone does not guarantee competition amongst constituent weights. More functionally relevant is a situation where weight enhancement in one group of inputs causes a corresponding weight reduction among others (Gilson and Fukai, 2011). To illustrate competition within the spike-based BCPNN weight structure, we selectively introduced pairwise correlation into the spike timings of 100 presynaptic cells. The correlated and uncorrelated input groups were stimulated to fire at the same rate (Figure 7A), so that the only difference in signal between neurons of the feedforward network (Figure 7B) was on the spike-timing level. Evolution of the weights was recorded for each connection (Figure 7C), and a specialized weight structure developed dependent upon the correlation coefficient *C* (Figure 7D). The difference between the distributions was calculated as the discriminability (Willshaw and Dayan, 1990):

(15) $$d^{\prime }=\frac{\mu_{+}-\mu_{0}}{\sigma_{0}}$$

The variable μ_+_ represented the mean of the correlated distribution, μ_0_ the mean of the uncorrelated distribution, and σ_+_ ≈ σ_0_ the standard deviation shared by the two distributions. The equilibrium weight distribution shifted proportionally for differing amounts of *C* (Figure 7E). As expected from a competitive mechanism, correlated neurons remained more potentiated beyond τ_*p*_ despite underlying long-term stabilizing pressures (see Figure 6). To assess the level of competition, we summed the synaptic weights for both the correlated and uncorrelated subpopulations for increasing *C*. As the weights stemming from the correlated population increased with *C*, the weights in the uncorrelated population decreased in response, while total weight values were kept relatively steady (Figure 7F). Furthermore, competition was reduced by increasing τ_*zi*_ and τ_*zj*_, which decreased the standard deviation of the terminal weight distribution and reduced the importance of each individual spike (Figure 7G).

### Intrinsic generation of graded persistent activity as a functional consequence of β

**Figure 8:**
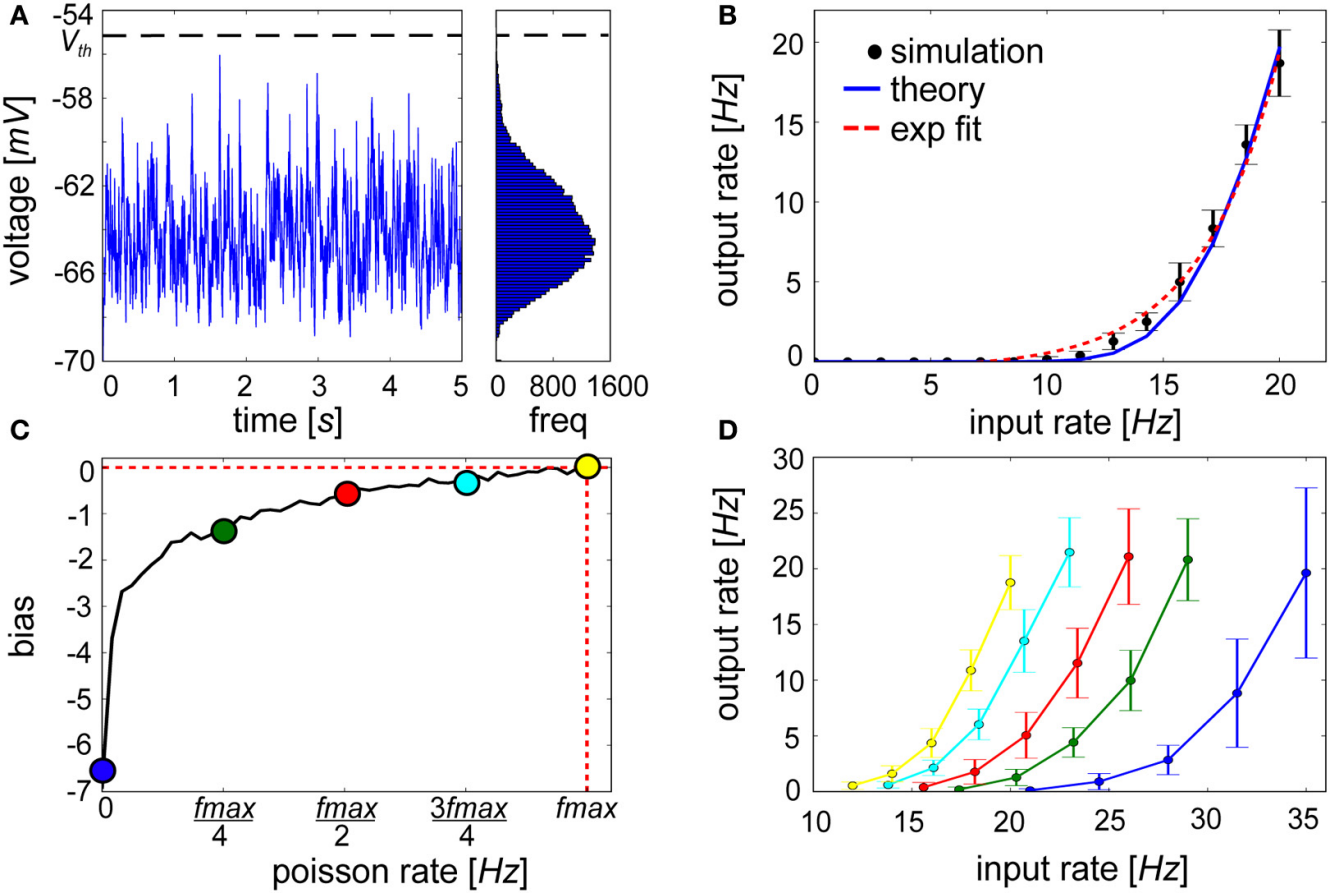
**Exponential activation function of a lowly firing IAF neuron is shifted by an injection of a hyperpolarizing current proportional to β_j_. (A)** Voltage trace and resulting long tail distribution of a membrane potential histogram from an IAF neuron approaching firing threshold of −55 mV (bin size = 0.15 mV). **(B)** The input-output curve of an IAF neuron with 30 inputs each firing at values listed along the abscissa (black, simulated; blue, see Materials and Methods for theoretical IAF rate). For low firing frequencies at or below 20 Hz, the function is approximately exponential (red-dotted fit: *y* = 0.48*e*^0.29(*x* − 7.18)^ − 0.47). **(C)** The bias term shows logarithmically increasing firing rate values of the neuron for which it is computed. **(D)** When hyperpolarizing current proportional to β_*j*_ is applied, neurons that have previously been highly active will be more easily excitable (e.g., yellow curve) compared to neurons that have had little recent history of firing (e.g., blue curve). Error bars depict the standard deviation gathered from 50 repeated experiments.

In spike-based BCPNN, output firing rates represent the posterior probability of observing a presented pattern. Although it is calculated by exponentiating the support activity (Equation 6), exponential input-output curves are rarely measured in experiments despite the apparent computational benefits of non-linear input transformation at the level of single neurons (Koch, 2004). To account for these biological constraints, an alternative scenario is considered in which a neuron is stimulated by excitatory Poisson background input such that the mean voltage of its membrane potential is subthreshold (Figure 8A) and it fires up to intermediate levels. This background-driven regime enables spike production due to fluctuations in subthreshold membrane voltage, and is thought to approximate *in vivo* conditions during which cortical neurons are bombarded by ongoing synaptic background activity (Destexhe et al., 2001).

We found that linearly increasing the level of presynaptic drive in the presence of background activity caused an expansive non-linearity in the IAF input-output curve within a physiologically relevant <1 up to 20 Hz range of firing, which has been reported previously for conductance-based IAF neurons (Fourcaud-Trocmé et al., 2003) and cortical neurons (Rauch et al., 2003). The time-averaged firing rate was well-approximated by an exponential function (Figure 8B). Relating back to Figure 1, information deemed relevant in the form of increased activity by a subset of presynaptic sources can cause the postsynaptic neuron to ascend its activation function. Inhibitory drive could dominate if other active presynaptic neurons signaled counter-evidence. Although they are excluded here, such interactions would not elicit a qualitative deviation in the input-output curve from Figure 8B.

Although functional synaptic aspects have been emphasized up until this point, a distinct role for intrinsic plasticity was not precluded. The neural input-output relationship is controlled by the abundance, kinetics, and biochemical properties of ion channels present in the postsynaptic cell membrane. This is represented in spike-based BCPNN by the variable β_*j*_, which is a function of the prior probability of postsynaptic activity *P_j_* (Equation 9, see Figure 8C), and quantifies a general level of excitability and spiking for the postsynaptic neuron. Because β_*j*_ → log(ϵ) for minimal and β_*j*_ → log(1) = 0 for maximal postsynaptic firing rates, β_*j*_ essentially lowered the probability for neurons that were seldom active previously to be driven passed threshold in the future. With regards to the statistical inference process, this excitability represents an *a priori* estimate of postsynaptic activation. The intuition is if an event is experienced for the first time, it will still be highly unexpected. To account for these effects neurally, β_*j*_ was treated as a hyperpolarizing current, *I*_β *j*_, that was continuously injected into the IAF neuron according to Equation 11.

The outcome of this type of dynamic modification is illustrated in Figure 8D. The input-output curve was shifted depending on β_*j*_, and the same synaptic input caused differing output levels. Similarly, LTP-IE provides a priming mechanism that can sensitively tune membrane properties of a neuron in response to altered levels of incoming synaptic input (Cudmore and Turrigiano, 2004). The A-type K^+^ channel gates the outward flow of current in an activity-dependent manner prescribed by a logarithmic transformation of *P_j_* (Hoffman et al., 1997; Daoudal and Debanne, 2003; Jung and Hoffman, 2009). The decay of a Ca^2+^-activated non-specific cationic (CAN) current mediated by activation of transient receptor potential (TRP) channels (Petersson et al., 2011) is another candidate that is thought to play a role in these graded changes (Fransén et al., 2006). Mirroring the cascading trace levels that collectively compute β_*j*_, multiple time scales of TRP current decay rate have been identified including a fast decay of 10 ms (Faber et al., 2006), a medium decay of 200–300 ms (Wyart et al., 2005) and a slow decay of 2–3 s (Sidiropoulou et al., 2009).

Intrinsic excitability has been conjectured to serve as a memory substrate via locally stored information in the form of a neuron's activity history. Despite the lack of temporal specificity that exists for synapses, intrinsic effects provide an alternative computational device that is presumably beneficial for learning and memory. We therefore asked how β_*j*_ could account for functional aspects associated with the modulation of intrinsic excitability.

**Figure 9:**
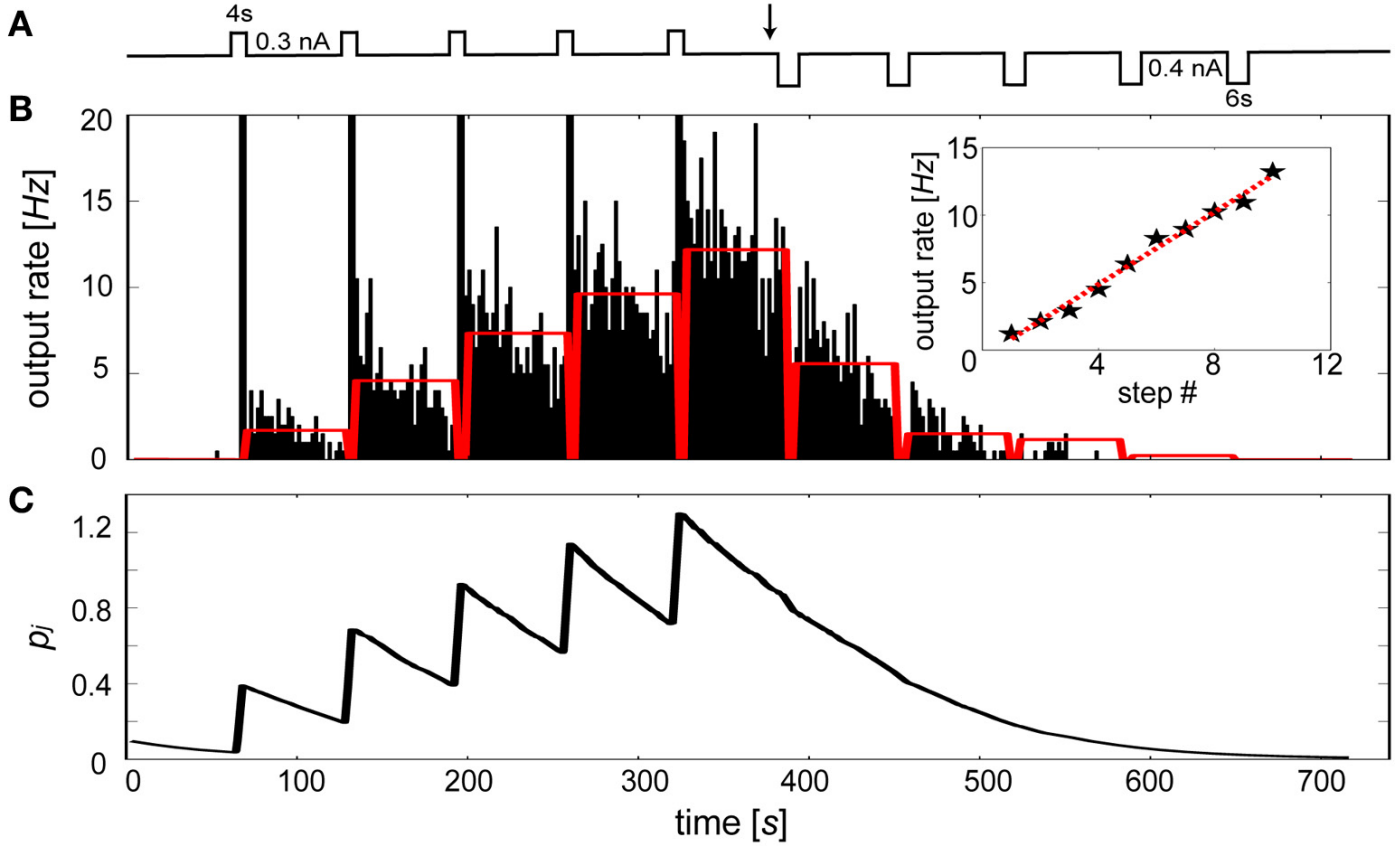
**The bias term reproduces the graded persistent activity found in entorhinal cortical neurons. (A)** Stimulation protocol. Repetitive depolarizing followed by hyperpolarizing current injections (switch occurs at black arrow) of the IAF neuron including β_*j*_. **(B)** Peristimulus time histogram (2 s bin width) of the elicited discharge. Red bars indicate the time averaged activity of each 1 min post-stimulus interval. Time averaged activity of 1 min post-stimulus intervals using 0.3 nA depolarizing steps each lasting 2 s (stars, red-dotted line: linear fit). **(C)** Underlying *P_j_* trace evolution during the simulation.

Specifically, we sought to model the rapid changes in intrinsic excitability found in slice preparations of layer V neurons from entorhinal cortex in rat (Egorov et al., 2002). In this study, initially silent neurons were repeatedly depolarized leading to a graded increases in their persistent firing levels. It was also shown that persistent activity states were deactivated by applying hyperpolarizing steps until quiescence. Figure 9A summarizes this stimulus protocol, which was applied to an *I*_β*j*_-modulated IAF neuron in the presence of background excitation. Duration and magnitude of the transient events were parameterized according to Egorov et al. (2002), using depolarizing steps of 0.3 nA for 4 s each and hyperpolarizing steps of 0.4 nA for 6 s each. The resulting activity of the neuron is illustrated by Figure 9B. Stable periods of elevated and suppressed firing rates were associated with increases and decreases in *I*_β*j*_, respectively. To achieve quantitatively similar graded persistent firing levels as was shown in Egorov et al. a τ_*p*_ of 60 s was used, similar to induction time courses observed for LTP-IE in neurons from visual cortex (Cudmore and Turrigiano, 2004) and cerebellar deep nuclear neurons (Aizenman and Linden, 2000). The sustained levels of activation were noisier than the *in vitro* preparation of Egorov et al., presumably due to the presence of excitatory synaptic background activity in the model.

Importantly, the increased rate of firing caused by each depolarizing stimulus application period led to a continuum of levels up to *f*_max_ Hz, rather than discretely coded activity levels (Fransén et al., 2006). The number of levels was arbitrary and depended on both the magnitude and duration of the pulse, displaying peak frequencies (<20 Hz) similar to those that were assumed for *f*_max_. To test this, ten depolarizing 2 s current steps were induced, producing a continuum of levels that was approximately linear with a regression coefficient of 1.33 (Figure 9B inset, red dotted line). Discharges were sustained by changes in the *P_j_* trace (Figure 9C). Each depolarizing step led to the generation of spikes which transiently increased *P_j_* and made β_*j*_ less negative. Conversely, each hyperpolarizing step tended to silence output activity, decreasing β_*j*_ and making it more difficult for the neuron to reach threshold. A bidirectional effect of β_*j*_ was apparent here, as excitability decreased when the neuron was depotentiated (Daoudal et al., 2002).

### Demonstrating probabilistic inference using a simple network

Up to this point, *w*_*ij*_ and β_*j*_ have been treated independently, but by virtue of a shared *P_j_*, this is not always the case in terms of network dynamics. A low excitability β_*j*_ for a historically inactive neuron would not necessarily detract from the informative content of the neuron *per se*, rather it must be considered in conjunction with its incoming weights *w*_*ij*_. It is entirely plausible that *w*_*ij*_ would be very high. In terms of the inference task, this would amount to neurons representing one specific class. To recapitulate previous examples, the feature “pink” might only signal the class “animal” if a flamingo was part of the training set, since such a distinctive feature is statistically rare yet easily classifiable.

**Figure 10:**
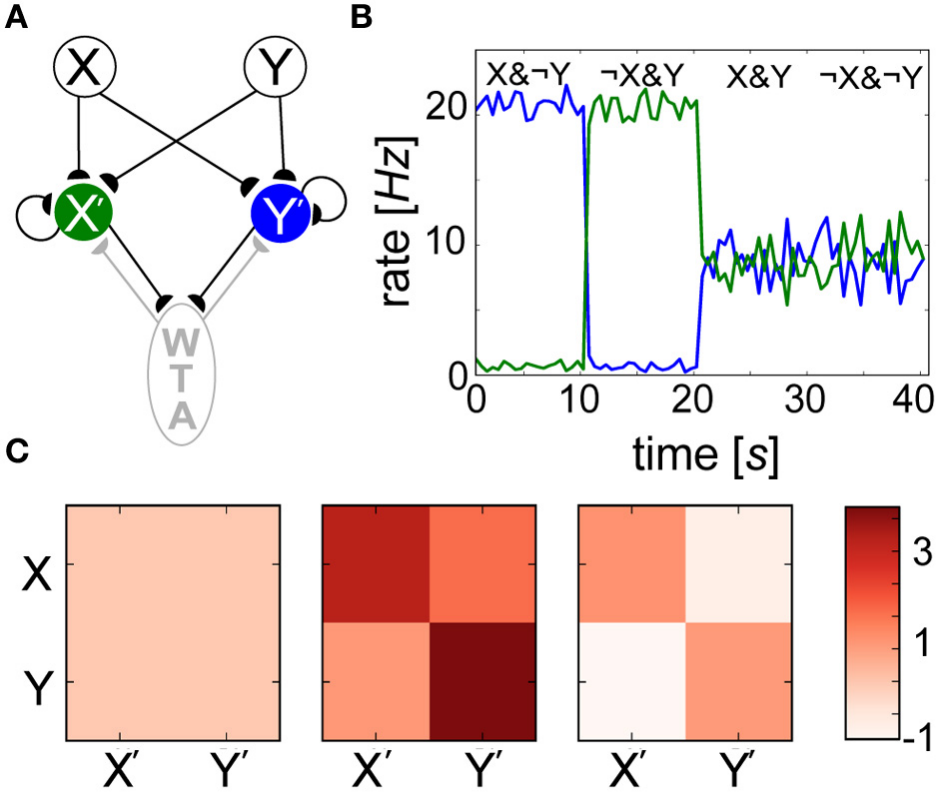
**Spiking BCPNN performs a simple Bayesian inference. (A)** Network architecture with excitatory (black) and inhibitory (gray) connections between local minicolumns. Input neurons of groups X and Y each project to the output layer X′ (green) and Y′ (blue), which mutually inhibit each other via an inhibitory WTA population (gray). **(B)** Posterior probability distributions are reflected by the output rates of postsynaptic neuron pools X′ and Y′ (colors corresponding to **A**) in 1 s bins during recall. **(C)** Evolution of the mean weight matrix during training, where each cell represents the averaged activity for all 900 connections. Three snapshots were taken during learning: one at the beginning, one a tenth of the way through, and one at the end of the simulation. Weights that were developed in alternating 200 ms intervals were initially volatile, but eventually settled into a symmetrical terminal weight structure.

Since neither weights *w*_*ij*_ nor biases β_*j*_ alone were able to reliably predict the outcome of learning, we introduced a simple network model (Figure 10A) to show how interwoven synaptic and nonsynaptic computations could perform a Bayesian inference task. Input layer minicolumns (X, Y) were all-to-all connected to the output layer (X′, Y′), each consisting of 30 neurons. In order to implement WTA, output layer neurons were recurrently connected amongst themselves (connection probability = 0.2), and reciprocally to an inhibitory population of 10 neurons (connection probability = 0.5). Ten seconds of alternating, orthogonal Poisson stimulation patterns (i.e., *f*_max_ or ϵ Hz) were applied to input layer groups and identically to their corresponding output groups. Over the course of training, specialized weights *w*_*ij*_ developed (Figure 10C) in which connections between X (Y) and X′ (Y′) increased in strength since they were coactive during training, and connections between X (Y) and Y′ (X′) decreased in strength since their activations were temporally disjoint. Since both X′ and Y′ were active for half of the training, their *P_j_* traces saturated at 0.5 (not shown). A simulation paradigm was employed in which weights were disabled during training (*g*_max_ = 0) and frozen after learning (κ = 0) for the sake of simplicity and since such effects have been hypothesized to mimic neuromodulatory interactions (Hasselmo, 1993).

The neurons weighed all available evidence and fired according to their inferred Bayesian weights and biases, and levels of uncertainty in these patterns were represented by neuronal firing rates during recall (Figure 10B). Recall could be performed irrespective of the stimulus duration, which was simply chosen to match τ_*p*_ here. For the trivial cases in which output neurons were presented with the exact input stimulus pattern received during training (X&¬Y, X&¬Y), certainty was exhibited by firing rates approaching *f*_max_ = 20 Hz. The reciprocal readout neurons in these scenarios had a lower level of belief in the incoming patterns due to the inhibition that developed between both sets of anti-correlated groups during training. In more interesting scenarios, output layer neurons displayed intermediate firing rates when both input populations were active (X&Y) due to inhibition of the novel pattern, and responded with uncertainty without any input pattern (¬X&¬Y), as their activity was dominated by β_*j*_ in the absence of presynaptic input. WTA ensured that either group X′ or Y′ temporarily won, or fired at *f*_max_ Hz while their counterpart was silent. This meant in both cases (X&Y) and (¬X&¬Y), neurons tended to fire on average at ${}^{\frac{f_{\text{max}}}{2}}$ Hz in this simple example.

## Discussion

That the brain could encode probabilistic models is a radical departure from classical approaches in neuroscience, which assume a bottom–up mechanistic view of computational units as input filters. Nevertheless, given that both human behavior in psychophysical tasks (Wolpert and Körding, 2004; Knill, 2005; Tassinari et al., 2006) and recorded neural activity in different brain areas (Carpenter and Williams, 1995; Rao and Ballard, 1999; Yang and Shadlen, 2007; Summerfield and Koechlin, 2008; Berkes et al., 2011; D'Acremont et al., 2013) have been shown to be able to carry out probabilistic operations, it has been suggested that a Bayesian coding hypothesis may be a generic property of neural computation. Models have been devised to show how Bayesian inference could be carried out by neurons and/or their networks, demonstrating various levels of neurobiological realism and capturing several general properties thought to be relevant for information processing. Here, we have reconciled several of these properties by showing that the extension of BCPNN to the domain of spiking neurons enables a rich collection of dynamics that collectively approximate probabilistic inference.

### Interpretation of positive and negative synaptic weights in the model

Weights in the proposed model can switch between positive and negative values, such that an excitatory synapse may become inhibitory and vice-versa. A monosynaptic excitatory connection with conductance determined by the positive component of *w*_*ij*_ could exist in parallel with a disynaptic inhibitory connection set by the negative component. Evidence for this putative feedforward inhibitory microcircuit has been shown to be associated with postsynaptic spike rate (Mathews and Diamond, 2003; Mori et al., 2004) or interneuron bypassing (Ren et al., 2007). Upon observing evidence that does not support the *a priori* belief level, the efficacy of synaptic transmission to excitatory sources via inhibitory interneurons neurons would increase, indirectly creating a net inhibitory drive. A direct channel would be preferred when the neuron is highly certain regarding the statistics of its input, so that the net effect would instead be excitatory. Since plastic weights turn negative, our model also implicitly assumes the presence of inhibitory plasticity (Kullmann et al., 2012), which has been previously investigated in the context of this disynaptic feedforward configuration (Vogels et al., 2011).

### Biological correlates

Plastic changes within biological memory systems are temporally dynamic phenomena, and arise as a result of biochemical cascades that are hierarchically coupled together at the molecular level. Despite this, and not least for reasons of computational convenience, phenomenological models of plasticity implicitly neglect both the contribution of the underlying biochemical pathways to the overarching computation along with their wide ranging timescales of operation. Furthermore, there is typically no explicit representation of memory age, thus rendering it impossible to take into account the relative familiarity of young or old memories. In contrast, our model explicitly implements the palimpsest property: three simple first order linear ordinary differential equations acting as temporally heterogeneous memory traces jointly serve the roles of assessing the novelty of the presented pattern on-line and estimating the relative probabilities used to perform inference (Sandberg et al., 2002).

The functional outcome of cascading memory traces at the synaptic level was a correlative Hebbian learning window with shape and relative width determined by τ_*zi*_ and τ_*zj*_. Preference for a left- or right-shifted temporal window has been shown in different experimental preparations (Froemke and Dan, 2002; Testa-Silva et al., 2010), and it is thought that temporal asymmetry may be attributable to the differential induction of NMDA-mediated LTP (Abbott and Blum, 1996). Strong connections could develop between pools of neurons in a directionally specific manner (Abeles, 1991) during a training period of externally applied input (Sompolinsky and Kanter, 1986). Stored patterns could then be sequentially recalled forwards or backwards through time depending on whether τ_*zi*_ > τ_*zj*_ or τ_*zi*_< τ_*zj*_.

Associative learning typically leads to runaway excitation or quiescence in the network context. There are modifications of learning rules that maintain stability, such as STDP models with multiplicative dependence of the change in weight on the strength of the synapse (van Rossum et al., 2000), which produce experimentally motivated unimodal equilibrium weight distributions (Song et al., 2005). Competition between synapses can be achieved using terms that account for activity dependent scaling (van Rossum et al., 2000), intermediate STDP rule parameterizations (Gütig et al., 2003), or a tuned STDP rule to fit a long-tailed weight distribution (Gilson and Fukai, 2011). Spike-based BCPNN demonstrates coexisting competition and stability that emerge from the statistical assumptions accompanying Bayesian weight updating. Such alternatives are relevant given increasing questions surrounding the ubiquity (Abbott and Nelson, 2000), fidelity (Lisman and Spruston, 2010) and precision (Kempter and Gerstner, 2001; Babadi and Abbott, 2010) of asymmetrical STDP as a generic biological learning rule.

One hypothesis for how stability can be achieved by neural circuits is that Ca^2+^ sensor pathways homeostatically regulate receptor trafficking to keep neuronal firing rates within a preferred regime (Rutherford et al., 1998; Turrigiano et al., 1998). Although spike-based BCPNN exhibited Hebbian synaptic plasticity, a regulatory mechanism arose that was able to both stabilize network activity and preserve existing memories. Activity could remain stable despite correlation-based changes in synaptic strength, and weights could be scaled down in a competitive manner when subsets of neurons were potentiated (Figures 6, 7). Thus, relative differences in synaptic efficacies could be preserved, similar to what is to be expected from synaptic scaling. This activity-dependent homeostatic mechanism is not unique to excitatory synapses. In spike-based BCPNN, negative *w*_*ij*_ increased when pre- and postsynaptic neurons were weakly active (Figure 4), which was justified from a probabilistic point of view. Given the interpretation of negative weights (see Interpretation of Positive and Negative Synaptic Weights in the Model), similar behavior would be expected due to an antagonistic upregulation of activity as a result of inhibitory synaptic scaling targeting pyramidal cells (Kilman et al., 2002).

Shared synaptic and nonsynaptic *P* traces in spike-based BCPNN suggest a novel probabilistic role for the integration of neural activity arising from molecular processes. Since the *P_j_* trace appears in the computation of both β_*j*_ and *w*_*ij*_, the model predicts coexpression of LTP/LTD and LTP-IE due to shared intracellular postsynaptic Ca^2+^ signaling cascades (Tsubokawa et al., 2000; Zhang and Linden, 2003). Indeed, LTP-IE is thought to share many common induction and expression pathways with LTP/LTD (Daoudal and Debanne, 2003), and experimental protocols used to study synaptic plasticity have often been shown to incidentally give rise to LTP-IE (Bliss and Lomo, 1973; Aizenman and Linden, 2000; Daoudal et al., 2002). As in LTP/LTD, LTP-IE is rapidly induced and long-lasting (Aizenman and Linden, 2000; Cudmore and Turrigiano, 2004), consistent with the notion of τ_*p*_.

### Related work

Several previous approaches have represented probabilities explicitly or intermediately using measures of neural activity. Compelling models have been proposed based on probabilistic population coding (Ma et al., 2006), where the variability within a population response encodes uncertainty in the stimulus, and belief propagation (Rao, 2005; Litvak and Ullman, 2009; Steimer et al., 2009), in which relevant states are estimated using internodal communication of messages that are alternatingly summed and multiplied over factor graphs. Linking a probabilistic modeling approach with multiple synergistic biological processes has recently been emphasized. Coupled synaptic plasticity and synaptic scaling (Keck et al., 2012) along with coupled STDP and homeostatic intrinsic excitability (Nessler et al., 2013) have been proposed in the context of the expectation maximization algorithm, whereas a model with coupled synaptic and intrinsic plasticity has been implemented using Gibbs sampling (Savin et al., 2014). This approach adopts a different machine learning-inspired algorithm, namely the naïve Bayes classifier. Despite its underlying independence assumptions, Naïve Bayes is known to perform surprisingly well in machine learning tasks compared with other advanced methods (Langley et al., 1992), and it is a subject of future work to develop biologically motivated benchmarks for these approaches in the domain of spiking neuronal networks.

Spike-based BCPNN was not intended to phenomenologically describe neurophysiological results. Rather, these similarities arise naturally from theoretically and biologically constrained assumptions. Learning in our model is based on three consecutively-fed traces that were temporally compatible with the signaling cascades of cellular processes underlying the induction of LTP and LTP-IE, and allowed each one to play a unique computational role during the online estimation of probabilities. Including multiple time scales in an attempt to more accurately capture the wide variety of molecular processes involved in memory has also been argued for in previous models (Fusi et al., 2005; Clopath et al., 2008). Another model hypothesized a memory scheme whereby LTP and LTP-IE could interact (Janowitz and van Rossum, 2006), but updates were asynchronous, which is difficult to reconcile with the coordinated interdependence known from biology (Daoudal and Debanne, 2003) and shown here for spike-based BCPNN.

Bayesian learning rules typically introduce rather specific assumptions about the makeup of activity or connectivity in the underlying neural circuit, and the one presented here introduces topological structure in the form of a WTA hypercolumn microcircuit. As for our model, this has previously been achieved by lateral inhibition (Nessler et al., 2013). In others, similar conditions were fulfilled by homeostatic intrinsic excitability (Habenschuss et al., 2012) and feedforward inhibition (Keck et al., 2012). Here, WTA normalizes outputs based on Equation 6 so that approximated posterior probabilities never exceed 1 within a hypercolumn. In biology, this normalization could be mediated by basket cell inhibition between local neural populations, a generic motif thought to be fundamental to cortical network organization (Douglas and Martin, 2004).

In spike-based BCPNN, such local neural populations, i.e., minicolumns, represent stochastic computational units. The probability of an event is reflected by the probability that its corresponding neurons spike during a given time step. Such considerations are advantageous from the perspective of neuromorphic hardware, in which Poisson-like noise and trial-to-trial variability physically manifest themselves as electronic phenomena. In the same vein, neural sampling (Buesing et al., 2011; Pecevski et al., 2011) has been proposed in which relevant computational units are not ensembles or columns of neurons but rather the stochastically firing neurons themselves. In both of these approaches, each spike carries a semantic interpretation. Several other models also take this viewpoint for spikes, and moreover utilize these input spikes for learning (Denève, 2008b; Nessler et al., 2013). In our model, the presence of a spike during a given time step signified an increase in confidence that the participating neurons are part of the presented pattern. The conductance-based neuron model we used is relatively detailed considering its alternatively proposed interpretation in terms of latent probabilistic operations, although IAF dynamics have been exploited elsewhere in this context (Denève, 2008a).

Care was taken to ensure that extension of spike-based BCPNN did not deviate from previous abstract implementations (Lansner and Ekeberg, 1989; Lansner and Holst, 1996). In doing so, the model here provides a direct way of exploring the spiking dynamics of systems in which BCPNN has been implicated, including neocortex (Sandberg et al., 2003; Johansson and Lansner, 2007; Lansner et al., 2013) and basal ganglia (Berthet et al., 2012). Such a step is necessary toward the goal of linking detailed neural mechanisms with complex probabilistic computations. Our approach can naturally be extended to the recurrent setting using the attractor memory paradigm, considered one of the most powerful tools for describing non-linear network dynamics (Lansner, 2009) yet notably absent thus far in the context of spiking models that incorporate probabilistic learning and inference.

In summary, we have described how a simple microcircuit comprised of intrinsically excitable conductance-based IAF neurons, interconnected by synapses endowed with correlative weight-dependent Hebbian-Bayesian plasticity, could readily approximate Bayesian computation. Spike-based BCPNN proposes a novel way of linking biochemical processes at the subcellular level and Poisson-like variability at the neuron level with complex probabilistic computations at the microcircuit level. It implies that the presence of a spike, or lack thereof, not only enacts measurable changes in the biochemical makeup of synapses and cells, but moreover contributes to an underlying, ongoing inference process.

### Conflict of interest statement

The authors declare that the research was conducted in the absence of any commercial or financial relationships that could be construed as a potential conflict of interest.

## References

[B1] Abbott L. F. Blum K. I. (1996). Functional significance of long-term potentiation for sequence learning and prediction. Cereb. Cortex 6, 406–416 10.1093/cercor/6.3.406 8670667

[B2] Abbott L. F. Nelson S. B. (2000). Synaptic plasticity: taming the beast. Nat. Neurosci. 3, 1178–1183 10.1038/81453 11127835

[B3] Abeles M. (1991). Corticonics: Neural Circuits of the Cerebral Cortex. New York, NY: Cambridge University Press 10.1017/CBO9780511574566

[B4] Abraham W. C. (2003). How long will long-term potentiation last? Phil. Trans. R. Soc. Lond. B 358, 735–744 10.1098/rstb.2002.1222 12740120 PMC1693170

[B5] Aizenman C. D. Linden D. J. (2000). Rapid, synaptically driven increases in the intrinsic excitability of cerebellar deep nuclear neurons. Nat. Neurosci. 3, 109–111 10.1038/72049 10649564

[B6] Babadi B. Abbott L. F. (2010). Intrinsic stability of temporally shifted spike-timing dependent plasticity. PLoS Comput. Biol. 6:e10009601 10.1371/journal.pcbi.1000961 21079671 PMC2973812

[B7] Bathellier B. Ushakova L. Rumpel S. (2012). Discrete neocortical dynamics predict behavioral categorization of sounds. Neuron 76, 435–449 10.1016/j.neuron.2012.07.008 23083744

[B8] Berkes P. Orbán G. Lengyel M. Fiser J. (2011). Spontaneous cortical activity reveals hallmarks of an optimal internal model of the environment. Science 331, 83–87 10.1126/science.1195870 21212356 PMC3065813

[B9] Berthet P. Hellgren-Kotaleski J. Lansner A. (2012). Action selection performance of a reconfigurable basal ganglia inspired model with Hebbian – Bayesian Go-NoGo connectivity. Front. Behav. Neurosci. 6:65 10.3389/fnbeh.2012.00065 23060764 PMC3462417

[B10] Bi G.-Q. Poo M.-M. (1998). Synaptic modifications in cultured hippocampal neurons: dependence on spike timing, synaptic strength, and postsynaptic cell type. J. Neurosci. 18, 10464–10472 9852584 10.1523/JNEUROSCI.18-24-10464.1998 PMC6793365

[B11] Bliss T. V. P. Collingridge G. L. (1993). A synaptic model of memory: long-term potentiation in the hippocampus. Nature 361, 31–39 10.1038/361031a0 8421494

[B12] Bliss T. V. P. Lomo T. (1973). Long-lasting potentiation of synaptic transmission in the dentate area of the anesthetized rabbit following stimulation of the perforant path. J. Neurophysiol. 232, 331–356 4727084 10.1113/jphysiol.1973.sp010273 PMC1350458

[B13] Boerlin M. Denève S. (2011). Spike-based population coding and working memory. PLoS Comput. Biol. 7:e1001080 10.1371/journal.pcbi.1001080 21379319 PMC3040643

[B14] Buesing L. Bill J. Nessler B. Maass W. (2011). Neural dynamics as sampling: a model for stochastic computation in recurrent networks of spiking neurons. PLoS Comput. Biol. 7:e1002211 10.1371/journal.pcbi.1002211 22096452 PMC3207943

[B15] Carpenter R. H. Williams M. L. (1995). Neural computation of log likelihood in control of saccadic eye movements. Nature 377, 59–62 10.1038/377059a0 7659161

[B16] Clopath C. Ziegler L. Vasilaki E. Buesing L. Gerstner W. (2008). Tag-trigger-consolidation: a model of early and late long-term-potentiation and depression. PLoS Comput. Biol. 4:e1000248 10.1371/journal.pcbi.1000248 19112486 PMC2596310

[B17] Cudmore R. H. Turrigiano G. G. (2004). Long-term potentiation of intrinsic excitability in LV visual cortical neurons. J. Neurophysiol. 92, 341–348 10.1152/jn.01059.2003 14973317

[B18] D'Acremont M. Fornari E. Bossaerts P. (2013). Activity in inferior parietal and medial prefrontal cortex signals the accumulation of evidence in a probability learning task. PLoS Comput. Biol. 9:e1002895 10.1371/journal.pcbi.1002895 23401673 PMC3561043

[B19] Daoudal G. Debanne D. (2003). Long-term plasticity of intrinsic excitability: learning rules and mechanisms. Learn. Mem. 10, 456–465 10.1101/lm.64103 14657257

[B20] Daoudal G. Hanada Y. Debanne D. (2002). Bidirectional plasticity of excitatory postsynaptic potential (EPSP)-spike coupling in CA1 hippocampal pyramidal neurons. Proc. Natl. Acad. Sci. U.S.A. 99, 14512–14517 10.1073/pnas.222546399 12391303 PMC137914

[B21] Denève S. (2008a). Bayesian spiking neurons I: inference. Neural Comput. 117, 91–117 10.1162/neco.2008.20.1.91 18045002

[B22] Denève S. (2008b). Bayesian spiking neurons II: learning. Neural Comput. 145, 118–145 10.1162/neco.2008.20.1.118 18045003

[B23] Destexhe A. Rudolph M. Fellous J.-M. Sejnowski T. J. (2001). Fluctuating synaptic conductances recreate *in vivo*-like activity in neocortical neurons. Neuroscience 107, 13–24 10.1016/S0306-4522(01)00344-X 11744242 PMC3320220

[B24] Douglas R. J. Martin K. A. C. (2004). Neuronal circuits of the neocortex. Annu. Rev. Neurosci. 27, 419–451 10.1146/annurev.neuro.27.070203.144152 15217339

[B25] Doya K. Ishii S. Pouget A. Rao R. P. N. (2007). Bayesian Brain: Probabilistic Approaches to Neural Coding. Cambridge, MA: MIT Press

[B26] Egorov A. V. Hamam B. N. Fransén E. Hasselmo M. E. Alonso A. A. (2002). Graded persistent activity in entorhinal cortex neurons. Nature 420, 173–178 10.1038/nature01171 12432392

[B27] Faber E. S. L. Sedlak P. Vidovic M. Sah P. (2006). Synaptic activation of transient receptor potential channels by metatropic glutamate receptors in the lateral amygdala. Neuroscience 137, 781–794 10.1016/j.neuroscience.2005.09.027 16289832

[B28] Florian R. V. (2007). Reinforcement learning through modulation of spike-timing-dependent synaptic plasticity. Neural Comput. 19, 1468–1502 10.1162/neco.2007.19.6.1468 17444757

[B29] Fourcaud-Trocmé N. Hansel D. van Vreeswijk C. Brunel N. (2003). How spike generation mechanisms determine the neuronal response to fluctuating inputs. J. Neurosci. 23, 11628–11640 14684865 10.1523/JNEUROSCI.23-37-11628.2003 PMC6740955

[B30] Fransén E. Tahvildari B. Egorov A. V. Hasselmo M. E. Alonso A. A. (2006). Mechanism of graded persistent cellular activity of entorhinal cortex layer v neurons. Neuron 49, 735–746 10.1016/j.neuron.2006.01.036 16504948

[B31] Frey U. Morris R. G. M. (1997). Synaptic tagging and long-term potentiation. Nature 385, 533–536 10.1038/385533a0 9020359

[B32] Froemke R. C. Dan Y. (2002). Spike-timing-dependent synaptic modification induced by natural spike trains. Nature 416, 433–438 10.1038/416433a 11919633

[B33] Fukunaga K. Stoppini L. Miyamoto E. Muller D. (1993). Long-term potentiation is associated with an increased activity of Ca2+/calmodulin-dependent protein kinase II. J. Biol. Chem. 268, 7863–7867 8385124

[B34] Fusi S. Drew P. J. Abbott L. F. (2005). Cascade models of synaptically stored memories. Neuron 45, 599–611 10.1016/j.neuron.2005.02.001 15721245

[B35] Gerstner W. (1995). Time structure of the activity in neural network models. Phys. Rev. E 51, 738–758 10.1103/PhysRevE.51.738 9962697

[B36] Gewaltig M.-O. Deismann M. (2007). NEST (NEural Simulation Tool). Scholarpedia. 2:1430 10.4249/scholarpedia.1430

[B37] Gilson M. Fukai T. (2011). Stability versus neuronal specialization for STDP: long- tail weight distributions solve the dilemma. PLoS ONE. 6:e25339 10.1371/journal.pone.0025339 22003389 PMC3189213

[B38] Gonon F. (1997). Prolonged and extrasynaptic excitatory action of dopamine mediated by d1 receptors in the rat striatum *in vivo*. J. Neurosci. 17, 5972–5978 9221793 10.1523/JNEUROSCI.17-15-05972.1997 PMC6573191

[B39] Gütig R. Aharonov R. Rotter S. Sompolinsky H. (2003). Learning input correlations through nonlinear temporally asymmetric hebbian plasticity. J. Neurosci. 23, 3697–3714 12736341 10.1523/JNEUROSCI.23-09-03697.2003 PMC6742165

[B40] Habenschuss S. Bill J. Nessler B. (2012). Homeostatic plasticity in bayesian spiking networks as expectation maximization with posterior constraints. Adv. Neural Inf. Process. Syst. 25, 782–790

[B41] Hasselmo M. E. (1993). Acetylcholine and learning in a cortical associative memory. Neural Comput. 5, 32–44 10.1162/neco.1993.5.1.32

[B42] Hoffman D. A. Magee J. C. Colbert C. M. Johnston D. (1997). K+ channel regulation of signal propagation in dendrites of hippocampal pyramidal neurons. Nature 387, 869–875 10.1038/42571 9202119

[B43] Izhikevich E. M. (2007). Solving the distal reward problem through linkage of STDP and dopamine signaling. Cereb. Cortex 17, 2443–2452 10.1093/cercor/bhl152 17220510

[B44] Janowitz M. K. van Rossum M. C. W. (2006). Excitability changes that complement Hebbian learning. Network 17, 31–41 10.1080/09548980500286797 16613793

[B45] Johansson C. Lansner A. (2007). Towards cortex sized artificial neural systems. Neural Netw. 20, 48–61 10.1016/j.neunet.2006.05.029 16860539

[B46] Jung S.-C. Hoffman D. A. (2009). Biphasic somatic A-Type K+ channel downregulation mediates intrinsic plasticity in hippocampal CA1 pyramidal neurons. PLoS ONE. 4:e6549 10.1371/journal.pone.0006549 19662093 PMC2717216

[B47] Keck C. Savin C. Lücke J. (2012). Feedforward inhibition and synaptic scaling – two sides of the same coin? PLoS Comput. Biol. 8:e1002432 10.1371/journal.pcbi.1002432 22457610 PMC3310709

[B48] Kempter R. Gerstner W. (2001). Intrinsic stabilization of output rates by spike-based hebbian learning. Neural Comput. 13, 2709–2741 10.1162/089976601317098501 11705408

[B49] Kempter R. Gerstner W. van Hemmen J. L. (1999). Hebbian learning and spiking neurons. Phys. Rev. E 59, 4498–4514 10.1103/PhysRevE.59.4498

[B50] Kilman V. van Rossum M. C. W. Turrigiano G. G. (2002). Activity deprivation reduces miniature IPSC amplitude by decreasing the number of postsynaptic GABAA receptors clustered at neocortical synapses. J. Neurosci. 22, 1328–1337 11850460 10.1523/JNEUROSCI.22-04-01328.2002 PMC6757564

[B51] Klopf H. A. (1972). Brain Function and Adaptive Systems- A Heterostatic Theory. Notes. Bedford, MA: Air Force Cambridge Research Laboratories Special Report

[B52] Knill D. C. (2005). Reaching for visual cues to depth: the brain combines depth cues differently for motor control and perception. J. Vis. 5, 103–115 10.1167/5.2.2 15831071

[B53] Knill D. C. Pouget A. (2004). The Bayesian brain: the role of uncertainty in neural coding and computation. Trends Neurosci. 27, 712–719 10.1016/j.tins.2004.10.007 15541511

[B54] Kobayashi K. Poo M.-M. (2004). Spike train timing-dependent associative modification of hippocampal CA3 recurrent synapses by Mossy Fibers. Neuron 41, 445–454 10.1016/S0896-6273(03)00873-0 14766182

[B55] Koch C. (2004). Biophysics of Computation: Information Processing in Single Neurons. New York, NY: Oxford University Press

[B56] Kuhn A. Aertsen A. Rotter S. (2003). Higher-order statistics of input ensembles and the response of simple model neurons. Neural Comput. 15, 67–101 10.1162/089976603321043702 12590820

[B57] Kuhn A. Aertsen A. Rotter S. (2004). Neuronal integration of synaptic input in the fluctuation-driven regime. J. Neurosci. 24, 2345–2356 10.1523/JNEUROSCI.3349-03.2004 15014109 PMC6729484

[B58] Kullmann D. M. Moreau A. W. Bakiri Y. Nicholson E. (2012). Plasticity of inhibition. Neuron 75, 951–962 10.1016/j.neuron.2012.07.030 22998865

[B59] Langley P. Iba W. Thompson K. (1992). An analysis of bayesian classifiers, in Proceedings of the Tenth National Conference on Artificial Intelligence, (San Jose, CA: MIT Press), 223–228

[B60] Lansner A. (2009). Associative memory models: from the cell-assembly theory to biophysically detailed cortex simulations. Trends Neurosci. 32, 178–186 10.1016/j.tins.2008.12.002 19187979

[B61] Lansner A. Ekeberg Ö. (1989). A one-layer feedback artificial neural network with a bayesian learning rule. Int. J. Neural Syst. 1, 77–87 10.1142/S0129065789000499

[B62] Lansner A. Holst A. (1996). A higher order bayesian neural network with spiking units. Int. J. Neural Syst. 7, 115–128 10.1142/S0129065796000816 8823623

[B63] Lansner A. Marklund P. Sikström S. Nilsson L.-G. (2013). Reactivation in working memory: an attractor network model of free recall. PLoS ONE. 8:e73776 10.1371/journal.pone.0073776 24023690 PMC3758294

[B64] Lisman J. (1989). A mechanism for the Hebb and the anti-Hebb processes underlying learning and memory. Proc. Natl. Acad. Sci. U.S.A. 86, 9574–9578 10.1073/pnas.86.23.9574 2556718 PMC298540

[B65] Lisman J. Spruston N. (2010). Questions about STDP as a general model of synaptic plasticity. Front. Synaptic Neurosci. 2:140 10.3389/fnsyn.2010.00140 21423526 PMC3059684

[B66] Litvak S. Ullman S. (2009). Cortical circuitry implementing graphical models. Neural Comput. 21, 3010–3056 10.1162/neco.2009.05-08-783 19686065

[B67] Ma W. J. Beck J. M. Latham P. E. Pouget A. (2006). Bayesian inference with probabilistic population codes. Nat. Neurosci. 9, 1432–1438 10.1038/nn1790 17057707

[B68] Markram H. Lübke J. Frotscher M. Sakmann B. (1997). Regulation of synaptic efficacy by coincidence of postsynaptic APs and EPSPs. Science 275, 213–215 10.1126/science.275.5297.213 8985014

[B69] Mathews G. C. Diamond J. S. (2003). Neuronal glutamate uptake contributes to GABA synthesis and inhibitory synaptic strength. J. Neurosci. 23, 2040–2048 12657662 10.1523/JNEUROSCI.23-06-02040.2003 PMC6742021

[B70] Miller K. D. Mackay D. J. C. (1994). The role of constraints in hebbian learning. Neural Comput. 6, 100–126 10.1162/neco.1994.6.1.100

[B71] Mori M. Abegg M. H. Gähwiler B. H. Gerber U. (2004). A frequency-dependent switch from inhibition to excitation in a hippocampal unitary circuit. Nature 431, 453–456 10.1038/nature02854 15386013

[B72] Mountcastle V. B. (1997). The columnar organization of the neocortex. Brain 120, 701–722 10.1093/brain/120.4.701 9153131

[B73] Nessler B. Pfeiffer M. Buesing L. Maass W. (2013). Bayesian computation emerges in generic cortical microcircuits through spike-timing-dependent plasticity. PLoS Comput. Biol. 9:e1003037 10.1371/journal.pcbi.1003037 23633941 PMC3636028

[B74] Nguyen P. V. Abel T. Kandel E. R. (1994). Requirement of a critical period of transcription for induction of a late phase of LTP. Science 265, 1104–1107 10.1126/science.8066450 8066450

[B75] Nordlie E. Gewaltig M.-O. Plesser H. E. (2009). Towards reproducible descriptions of neuronal network models. PLoS Comput. Biol. 5:e1000456 10.1371/journal.pcbi.1000456 19662159 PMC2713426

[B76] Pawlak V. Wickens J. R. Kirkwood A. Kerr J. N. D. (2010). Timing is not everything: neuromodulation opens the STDP gate. Front. Synaptic Neurosci. 2:146 10.3389/fnsyn.2010.00146 PMC3059689 21423532

[B77] Pecevski D. Buesing L. Maass W. (2011). Probabilistic inference in general graphical models through sampling in stochastic networks of spiking neurons. PLoS Comput. Biol. 7:e1002294 10.1371/journal.pcbi.1002294 22219717 PMC3240581

[B78] Peters A. Yilmaz E. (1993). Neuronal organization in area 17 of cat visual cortex. Cereb. Cortex 3, 49–68 10.1093/cercor/3.1.49 7679939

[B79] Petersson M. Yoshida M. Fransén E. (2011). Low-frequency summation of synaptically activated transient receptor potential channel-mediated depolarizations. Eur. J. Neurosci. 34, 578–593 10.1111/j.1460-9568.2011.07791.x 21777305 PMC3222851

[B80] Pfister J.-P. Dayan P. Lengyel M. (2010). Synapses with short-term plasticity are optimal estimators of presynaptic membrane potentials. Nat. Neurosci. 13, 1271–1275 10.1038/nn.2640 20852625 PMC3558743

[B81] Rao R. P. N. (2005). Hierarchical bayesian inference in networks of spiking neurons. Adv. Neural Inf. Process. Syst. 17, 1113–1120

[B82] Rao R. P. N. Ballard D. H. (1999). Predictive coding in the visual cortex: a functional interpretation of some extra-classical receptive-field effects. Nat. Neurosci. 2, 79–87 10.1038/4580 10195184

[B83] Rauch A. La Camera G. Lüscher H.-R. Senn W. Fusi S. (2003). Neocortical pyramidal cells respond as integrate-and-fire neurons to *in vivo* – like input currents. J. Neurophysiol. 90, 1598–1612 10.1152/jn.00293.2003 12750422

[B84] Ren M. Yoshimura Y. Takada N. Horibe S. Komatsu Y. (2007). Specialized inhibitory synaptic actions between nearby neocortical pyramidal neurons. Science 316, 758–761 10.1126/science.1135468 17478724

[B85] Roberts S. W. (1959). Control chart tests based on geometric moving averages. Technometrics 1, 239–250 10.1080/00401706.1959.10489860

[B86] Rutherford L. C. Nelson S. B. Turrigiano G. G. (1998). BDNF has opposite effects on the quantal amplitude of pyramidal neuron and interneuron excitatory synapses. Neuron 21, 521–530 10.1016/S0896-6273(00)80563-2 9768839

[B87] Sandberg A. Lansner A. Petersson K.-M. Ekeberg Ö. (2002). A Bayesian attractor network with incremental learning. Network 13, 179–194 10.1080/net.13.2.179.194 12061419

[B88] Sandberg A. Tegnér J. Lansner A. (2003). A working memory model based on fast Hebbian learning. Network 14, 789–802 10.1088/0954-898X/14/4/309 14653503

[B89] Savin C. Peter D. Lengyel M. (2014). Optimal recall from bounded metaplastic synapses: predicting functional adaptations in hippocampal area CA3. PLoS Comput. Biol. 10:e1003489 10.1371/journal.pcbi.1003489 24586137 PMC3937414

[B90] Schultz W. Peter D. Montague R. P. (1997). A neural substrate of prediction and reward. Science 275, 1593–1599 10.1126/science.275.5306.1593 9054347

[B91] Sidiropoulou K. Lu F.-M. Fowler M. A. Xiao R. Phillips C. Ozkan E. D. (2009). Dopamine modulates an mGluR5-mediated depolarization underlying prefrontal persistent activity. Nat. Neurosci. 12, 190–199 10.1038/nn.2245 19169252 PMC2727588

[B92] Soltani A. Wang X.-J. (2009). Synaptic computation underlying probabilistic inference. Nat. Neurosci. 13, 112–119 10.1038/nn.2450 20010823 PMC2921378

[B93] Sompolinsky H. Kanter I. (1986). Temporal association in asymmetric neural networks. Phys. Rev. Lett. 57, 2861–2864 10.1103/PhysRevLett.57.2861 10033885

[B94] Song S. Sjöström P. J. Reigl M. Nelson S. B. Chklovskii D. B. (2005). Highly nonrandom features of synaptic connectivity in local cortical circuits. PLoS Biol. 3:e68 10.1371/journal.pbio.0030068 15737062 PMC1054880

[B95] Steimer A. Maass W. Douglas R. J. (2009). Belief propagation in networks of spiking neurons. Neural Comput. 21, 2502–2523 10.1162/neco.2009.08-08-837 19548806

[B96] Stevenson I. H. Cronin B. Sur M. Kording K. P. (2010). Sensory adaptation and short term plasticity as bayesian correction for a changing brain. PLoS ONE. 5:e12436 10.1371/journal.pone.0012436 20865056 PMC2928744

[B97] Summerfield C. Koechlin E. (2008). A neural representation of prior information during perceptual inference. Neuron 59, 336–347 10.1016/j.neuron.2008.05.021 18667160

[B98] Tassinari H. Hudson T. E. Landy M. S. (2006). Combining priors and noisy visual cues in a rapid pointing task. J. Neurosci. 26, 10154–10163 10.1523/JNEUROSCI.2779-06.2006 17021171 PMC6674625

[B99] Testa-Silva G. Verhoog M. B. Goriounova N. A. Loebel A. Hjorth J. J. J. Baayen J. C. (2010). Human synapses show a wide temporal window for spike-timing-dependent plasticity. Front. Synaptic Neurosci. 2:12 10.3389/fnsyn.2010.00012 21423498 PMC3059666

[B100] Tetzlaff C. Kolodziejski C. Markelic I. Wörgötter F. (2012). Time scales of memory, learning, and plasticity. Biol. Cybern. 106, 715–726 10.1007/s00422-012-0529-z 23160712

[B101] Tsubokawa H. Offermanns S. Simon M. Kano M. (2000). Calcium-dependent persistent facilitation of spike backpropagation in the CA1 pyramidal neurons. J. Neurosci. 20, 4878–4884 10864945 10.1523/JNEUROSCI.20-13-04878.2000 PMC6772269

[B102] Turrigiano G. G. Leslie K. R. Desai N. S. Rutherford L. C. Nelson S. B. (1998). Activity-dependent scaling of quantal amplitude in neocortical neurons. Nature 391, 892–896 10.1038/36103 9495341

[B103] van Rossum M. C. W. Bi G.-Q. Turrigiano G. G. (2000). Stable hebbian learning from spike timing-dependent plasticity. J. Neurosci. 20, 8812–8821 11102489 10.1523/JNEUROSCI.20-23-08812.2000 PMC6773092

[B104] Vogels T. P. Sprekeler H. Zenke F. Clopath C. Gerstner W. (2011). Inhibitory plasticity balances excitation and inhibition in sensory pathways and memory networks. Science 334, 1569–1573 10.1126/science.1211095 22075724

[B105] Waelti P. Dickinson A. Schultz W. (2001). Dopamine responses comply with basic assumptions of formal learning theory. Nature 412, 43–48 10.1038/35083500 11452299

[B106] Willshaw D. Dayan P. (1990). Optimal plasticity from matrix memories: what goes up must come down. Neural Comput. 2, 85–90 10.1162/neco.1990.2.1.85

[B107] Wolpert D. M. Körding K. P. (2004). Bayesian integration in sensorimotor learning. Nature 427, 244–247 10.1038/nature02169 14724638

[B108] Wyart C. Cocco S. Bourdieu L. Léger J.-F. Herr C. Chatenay D. (2005). Dynamics of excitatory synaptic components in sustained firing at low rates. J. Neurophysiol. 93, 3370–3380 10.1152/jn.00530.2004 15673554

[B109] Yang T. Shadlen M. N. (2007). Probabilistic reasoning by neurons. Nature 447, 1075–1082 10.1038/nature05852 17546027

[B110] Yoshimura Y. Dantzker J. L. Callaway E. M. (2005). Excitatory cortical neurons form fine-scale functional networks. Nature 433, 868–873 10.1038/nature03252 15729343

[B111] Zhang W. Linden D. J. (2003). The other side of the engram: experience-driven changes in neuronal intrinsic excitability. Nat. Rev. Neurosci. 4, 884–900 10.1038/nrn1248 14595400

