# Supplementary material for: Synaptic and nonsynaptic plasticity approximating probabilistic inference
Source: Front Synaptic Neurosci. 2014 Apr 8;6:8. doi: 10.3389/fnsyn.2014.00008 (PMC3986567; doi:10.3389/fnsyn.2014.00008)
Supplement: Supplementary file 1 [file DataSheet1.PDF]

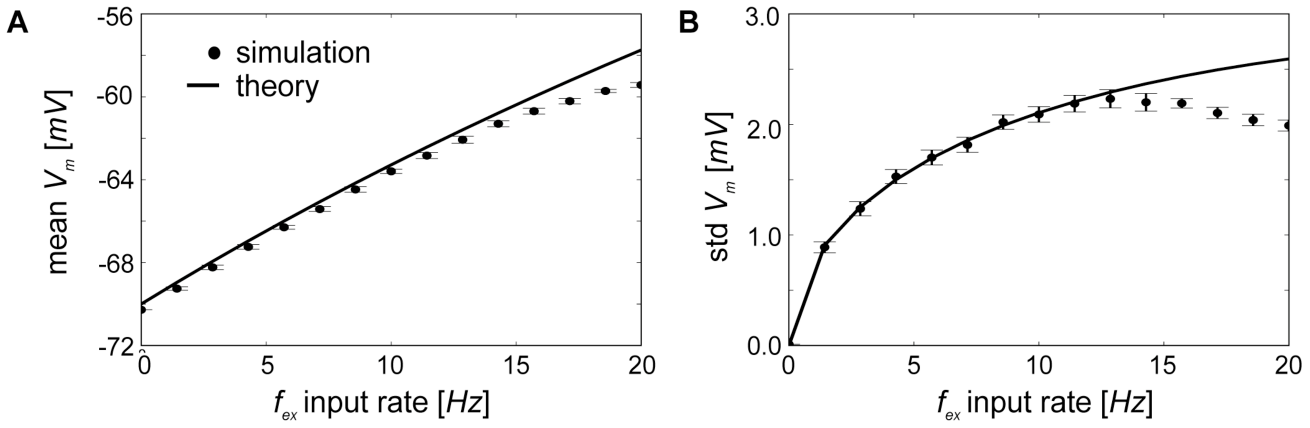

Figure S1. Measuring terminal V_m as a function of increasing synaptic input. **(A)** n_{ex}=30 Poisson input neurons with weights w_{ex} =10.75 elicit a linear increase in the mean and **(B)** a square root increase in the standard deviation of V_m. Solid lines represent the result of plotting Equation 10 directly (Kuhn, Aertsen and Rotter, 2004), and points represent the result of the NEST simulation using identical parameters. As the mean V_m is increasingly driven towards V_{th}=55 mV a deviation emerges around f_{ex}=13 Hz due to voltage reset in simulations, which distorts the shape of the terminal V_m distribution. Error bars depict the standard deviations after 50 simulations.

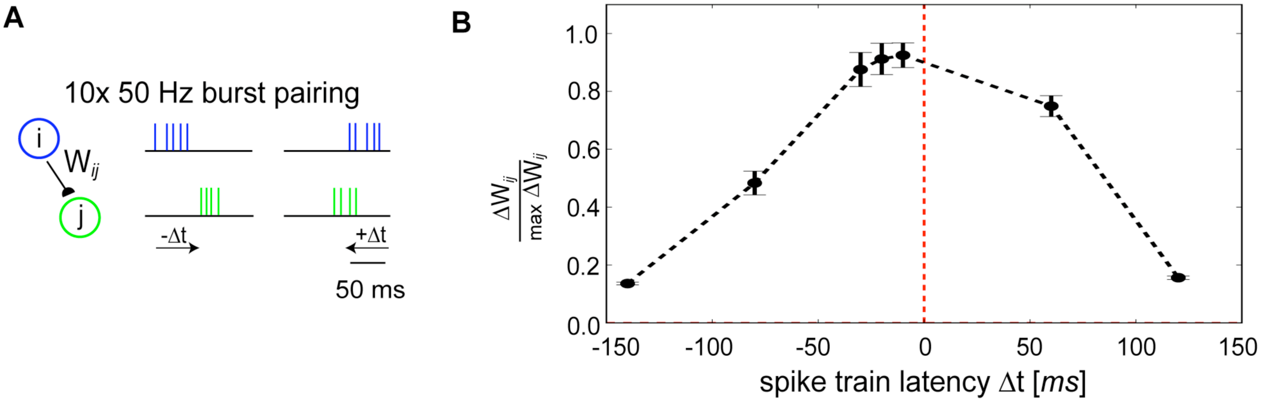

Figure S2. Spiking BCPNN potentiation magnitude depends on spike train overlap duration. **(A)** Schematic representation of the spike train-timing dependent plasticity conditioning protocol. Each pre (blue) - post (green) burst pairing is repeated 10 times at a frequency of 1 Hz for each time difference Δt = (-140, -80, -30, -20, -10, 60, 120) (Kobayashi and Poo, 2004). **(B)** Relative change in peak synaptic conductance for the BCPNN synapse using the parameters τ_{z_i} = 50 ms, τ_{z_j}= 50 ms, τ_e= 100 ms, and τ_p = 10000 ms. For each spike train, 5 pre- and 4 postsynaptic Poisson spike times were randomly generated in a 100 ms period (~50 Hz). Error bars represent standard deviation from 5 trials of randomly generated timings within the spike trains. Increased initial trace values de-emphasized the relevance of precise timings with respect to the overall magnitude of potentiation, as indicated by subtle differences between Δt = -30, -20 and -10.
